# Characterization of Parameter Uncertainty in Global Analysis for Ultrafast Spectroscopy Using Markov Chain Monte Carlo Sampling

**DOI:** 10.1021/prechem.5c00468

**Published:** 2026-03-27

**Authors:** Sullivan Bailey-Darland, Logan S. Lancaster, Taylor D. Krueger, Cheng Chen, Chong Fang

**Affiliations:** Department of Chemistry, 2694 Oregon State University, 153 Gilbert Hall, Corvallis, Oregon 97331, United States

**Keywords:** error analysis, parameter uncertainty, ultrafast spectroscopy, transient absorption, molecular dynamics, Markov chain Monte Carlo sampling

## Abstract

Ultrafast spectroscopy has advanced our understanding of chemical reaction mechanisms on molecular time scales, guiding the rational design of molecular systems and processes with spatial (atomic, nuclear) and temporal (electronic, vibrational) precisions. Kinetic analysis of ultrafast spectroscopic measurements yields valuable insights into the physical mechanisms that determine the behavior of a wide range of important chemical systems from chromophores and fluorescent proteins to nanosystems. One powerful and widely used method is global analysis, which uses a unified kinetic description of the entire spectrum. Importantly, interpretation of fit parameters to build a mechanistic understanding relies on an accurate estimate of the uncertainty. Software and numerical techniques using global analysis to fit data are well-developed; however, there is much less work on uncertainty analysis of the resulting kinetic parameters. Due to the nonlinear nature of modeling, traditional methods tend to underestimate the uncertainty. Building on previous work, we implement Markov chain Monte Carlo (MCMC) sampling to estimate the parameter uncertainty. We develop a procedure that can be readily included in the existing analysis software and demonstrate it on a series of generated and experimental femtosecond transient absorption data sets. Our results suggest that global analysis, using the simultaneously collected spectral data at many wavelengths, is crucial for accurate parameter estimation. The retrieved parameter uncertainty is well within 10% for a typical spectral data set with reasonable signal-to-noise ratios.

## Graphical abstract

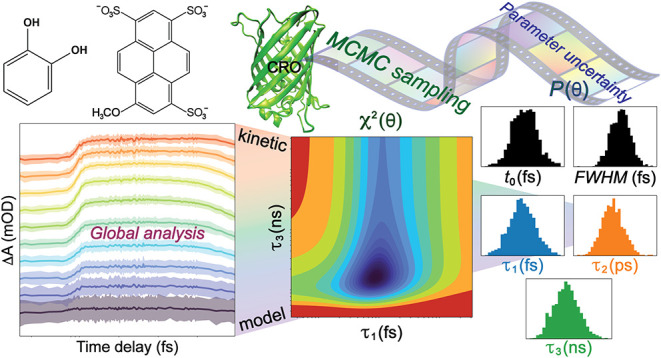

## Introduction

Ultrafast measurements on the femtosecond (fs) to picosecond (ps) time scales have revolutionized our understanding of light-driven chemical reactions with correlated atomic and electronic motions, which provide vivid details on miraculous natural phenomena like bioluminescence, light-harvesting, and vision.^1–4^ To extract physically meaningful information with ideally generalizable principles, researchers rely on robust modeling to interpret the complex spectroscopic data. Many models for time-resolved spectroscopy are based on the transiently generated and exponentially decaying populations, which can create measurable signals. These models assume first-order reaction kinetics for modeling the population change over time, a reasonable assumption for most systems. However, another pivotal feature of a model is how errors are introduced, estimated, and reported which has been treated as largely settled for transient absorption analysis for several decades. A classic example includes the calculation of radiative and nonradiative decay rates using the apparent excited-state lifetime and fluorescence quantum yield (FQY), wherein the small errors in parameters retrieved from ultrafast spectral data could lead to large uncertainty and/or ambiguous assignments.^5,6^ Recently, armed with the massively increased computing power, there have been works revisiting this topic^7–10^ with new avenues for interpreting complicated and overlapped spectra which have gained more interest and momentum across modern disciplines.

One foundational modeling technique is global analysis using various kinetic schemes, which analyzes the entire spectrum of measured wavelengths to more accurately determine the underlying kinetics.^11,12^ If successful, it allows experimental data to be deconvolved into a set of discrete populations with characteristic spectra and physically motivated dynamics. These states can then be identified with conformations or species that occur during the ultrafast processes. Much of the early work on computational data analysis was concerned with parameter uncertainty, but was constrained by the computational power available at the time. It is well-known that recovering parameters when fitting exponentials accurately can be difficult,^13,14^ which suggests that similar issues may arise in global analysis. Unlike linear least-squares fits, the parameter uncertainty can be complex and have large covariances between parameters, and estimates using traditional methods often underestimate the true uncertainty; therefore, more complex methods are needed to perform the uncertainty analysis,^15–17^ including Bayesian analysis beyond limitations set by the Fourier theory after removing the incoherent population term from time-dependent data.^9,10^

There have been many computational and numerical advances in global analysis modeling. Notable examples include the software packages Glotaran (and Pyglotaran), KiMoPack, and OPTIMUS, which have made significant advances in the speed and generalizability of this modeling.^12,18–20^ Open-source software has made data fitting much easier and more routine for spectroscopists. Nonetheless, recent computational advances focused primarily on ways to find the best-fit parameters and tended to neglect the analysis of parameter uncertainty. One exception is the method proposed to fit the data using Markov chain Monte Carlo (MCMC) sampling.^7^

In this work, we investigated which conditions control the uncertainty in kinetic parameters recovered from global analysis, and how the uncertainty affects and helps to evaluate the best fits. Building on the work conducted^7^ and inspired by analysis of complex models in other fields,^21^ we develop MCMC sampling methods to estimate the uncertainty and clarify the fit quality produced by global analysis. We extend this method to more complex systems and demonstrate its suitability on real experimental data sets. We implement this analysis as an addendum to global analysis (compatible with the widely used fitting software), setting two goals for this work. First, for the experimental spectroscopists, we aim to provide important insights into the experimental and modeling choices that can make data fitting easier and more robust. Second, we contribute a way to easily include MCMC sampling into existing global analysis fitting routines, which should avoid major changes to the existing analysis methods for an enhanced interpretation of key spectroscopic findings. Such a robust uncertainty analysis within the relevant parameter space provides deeper insights into light-sensitive molecular systems from an aromatic compound, a methoxylated photoacid to a fluorescent protein biosensor with increasing complexity and diverse functionalities.

## Results and Discussion

### Background and Assumptions

Transient absorption (TA) spectroscopy measures the difference in absorption between the excited and ground state species as a function of time and wavelength. Experimentally, the system can be excited by a pump pulse at time zero (*t*_0_), the light intensity of a subsequent probe pulse is then measured for the excited state *I*_1_ at time *t*. Using a reference measurement of the ground state *I*_0_, the resultant spectrum is given by $\Delta A\left(\lambda ,t\right)=\log\left(\frac{I_{0}(\lambda )}{I_{1}\left(\lambda ,t\right)}\right)$ .^4,14,22^ Since the excited-state species are transient, we expect *I*_1_ to approach *I*_0_ for long time delays and so Δ*A*(λ,*t*) eventually decays to zero.

Global analysis assumes that the difference spectra Δ*A*(λ,*t*) are due to a discrete set of transient species, each of which has a fixed emission/absorption profile and follows first-order kinetics. Crucially, it also assumes bilinearity, meaning that the signal can be written as a linear superposition of time-dependent spectra from individual species. Alternative methods with fewer constraints, such as lifetime density analysis^22^ and general curve-fitting methods like multivariate curve resolution (MCR)^14^ and singular-value decomposition (SVD) tend to have less assumptions, but require many more free parameters. In particular, global analysis has been enormously successful as a tool for ultrafast spectroscopy. Like many good scientific models, the assumptions it makes can achieve highly interpretable results without sacrificing much accuracy or broad applicability. This method allows spectroscopists to quickly come to physical insights from complex data sets by inspecting the intensity decay/rise and peak wavelength shifts. It is worth mentioning that there are a number of cases, particularly in ultrafast spectroscopy where the assumptions of global analysis may break down (see the discussion below for details). Even if the modeling is not perfect, in practice it is often the most convenient way to quickly draw useful conclusions from an experiment.^11,15,17^

### Model Description

Here we use the notation ψ­(λ,*t*) for the output of the model, to distinguish from the data Δ*A*(λ,*t*). Mathematically, for *N* species the time-resolved spectrum at each wavelength and time is

1 $$\psi \left(\lambda ,t\right)=\sum_{i=1}^{N}A_{i}\left(\lambda \right)X_{i}\left(t\right)$$

where *A*_*i*_(λ) is the spectrum of species *i* and *X*_*i*_(*t*) is the population of species *i* at time *t*. For any experiment with a finite set of wavelengths, this can be written as a matrix equation, Ψ = *AX*. This equation has notable degeneracy if one tries to just find best-fit matrices, as is done with SVD. For example, Ψ = *AX* = *ARR* ^–1^X = *A*′*X*′ for any matrix *R* such that *RR* ^–1^ is the identity. Global analysis approaches this problem by constraining the pertinent populations to be physically meaningful using a kinetic model, then finds the spectra in a way that best fits the data. Conversely, one could approach this problem by constraining the spectra (compare the “kinetic model” vs “spectral model”).^23^ In general the component spectra are unknown and contain rich or complex structure/features, while the relaxation dynamics, typically having a known form, are easier to parametrize than the spectra themselves; however, mathematically there is no preference.^12^

### Population Dynamics

The population dynamics, *i.e*., the curves *X*_*i*_(*t*), are determined by the kinetic model and a set of parameters describing the properties of the excitation pulse. The kinetic model describes which species are initially excited and how they relax to the electronic ground state. In the most general form, the dynamics could be described in the following way

2 $$\frac{dX_{i}}{dt}=\sum_{j=1}^{N}T_{\mathrm{ij}}X_{j}+J_{i}\;\mathrm{IRF}\left(t\right)$$

where IRF­(*t*) is the instrument response function (IRF) describing the excitation pulse intensity, and *J*_*i*_ determines whether *X*_*i*_ can be initially excited (*J*_*i*_ = 0 means it does not). The matrix *T*_*ij*_ is the “transfer matrix” between species; *T*_*ij*_ is the rate for species *j* converting to species *i*. More intuitively, the model is often described using a rate matrix *K*_*ij*_, where *K*_*ij*_ is the transition rate from *j* to *i* if *i* ≠ *j*, and *K*_*ii*_ is the decay of *i* to the ground state (*i.e*., resting state). The transfer and rate matrices are identical except for the diagonal, where *T*_*ii*_ = −∑_*j*=1_^*N*^ *K*_*ji*_ (the total decay from state *i*).

The IRF can be described in increasingly complex ways, depending on the time scale of the experiment and properties of the optical setup (*e.g*., ∼100 fs in a transient absorption apparatus powered by a Ti:sapphire regenerative laser amplifier with ∼35 fs output pulse duration).^24^ In most ultrafast spectroscopy experiments, the finite width of the laser pulse is needed to accurately describe dynamics on the time scale of the excitation pulse width,^5,25,26^ which can be parametrized as the full width at half-maximum (FWHM) of the pulse. This results in the IRF given by

3 $$\mathrm{IRF}\left(t\right)=\frac{1}{\sigma \sqrt{2\pi }}e^{-(1/2){\left(\left(t-t_{0}\right)/\sigma \right)}^{2}}$$

with $\mathrm{FWHM}=\sqrt{8\ln2}\sigma$ . This equation will be used for generating and fitting data sets for the rest of this work, and so IRF­(*t*) is determined if *t*_0_ and FWHM are known. Additional parameters can be introduced to fit experimental effects such as dispersion of the probe pulse and coherent artifacts, which can be done in many of the open-source software packages including Glotaran,^12^ as well as the wavelength dependence of the IRF. An alternative method can be used to remove chirp and/or the coherent artifacts before the analysis. While IRF­(*t*) describes the pulse intensity, its integral in this equation becomes one (see eq 3). The value of *J*_*i*_ in eq 2 can thus be considered as the actual concentration of the species produced after excitation. For simplicity, *J*_*i*_ is often set to zero or one. More advanced analyses could allow *J*_*i*_ to vary, if species are excited in different amounts such as inhomogeneous populations in a tree fungal pigment.^27^

Commonly, global analysis assumes first-order kinetics for all reactions in dilute samples,^11,12^ which greatly simplifies the equation and solution. These kinetics can be modeled using a kinetic matrix *K*_*ij*_ that characterizes the allowed transitions (see above). The system can be solved analytically for the curves *X*_*i*_(*t*) (details in the SI),^18^ so the dynamics are completely specified if *J*_*i*_, *t*_0_, FWHM, and *K*_*ij*_ are known. Generally, when fitting a data set the initial population *J*_*i*_ and structure of the matrix *K*_*ij*_ are specified. The result is that, given a specific kinetic model (*i.e*., defining states and allowed transitions), there is a small set of parameters that determine *X*_*i*_(*t*): the time zero (*t*_0_) and width (FWHM) of the excitation pulse and the set of rate constants *k*_*i*_ allowed in the kinetic model. By convention, we will use the lifetimes τ_*i*_ = 1/*k*_*i*_ rather than the rates. Our goal is to estimate the uncertainty of these parameters.

### Spectra for Each Species

The curves *A*_*i*_(λ) can be quickly identified as the spectra associated with each species, which have a variety of forms. The value of *A*_*i*_(λ) is in reference to a ground-state spectrum, and so the curve is actually the difference in absorption relative to the ground-state species. This can result in positive and/or negative features, depending on the system. Unlike the populations *X*_*i*_(*t*), the spectra *A*_*i*_(λ) are not parametrized.

### Fitting Procedure

When fitting, we consider the kinetic model to be constant: the number of species, the number of rate constants, and the initial excited population are fixed. The model has quite a few parameters that can be varied: (num. species) × (num. wavelength) values that appear in *A*_*i*_(λ) and (num. of rate constants + num. of IRF parameters) values that determine *X*_*i*_(*t*). However, the parameters do not all appear in the same way. The parameters in *A*_*i*_(λ) appear linearly and can be estimated rather straightforwardly if *X*_*i*_(*t*) are known, while the parameters that determine *X*_*i*_(*t*) appear nonlinearly. This is a separable optimization problem, and this method of solving is closely related to variable projection.^28^ For ease of notation, we will denote these nonlinear parameters as θ. Since the parameters can vary over multiple orders of magnitude, in particular the lifetimes from initial charge transfer/solvation on fs/ps time scales to fluorescence emission on nanosecond (ns) time scale, the nonlinear parameters are log-transformed before being used in any fitting algorithms. This can also be justified by noticing that most of the nonlinear parameters appear as exponents. More details on the exact implementation can be found in the Materials and Methods section (see below).

It is easiest to see how the best-fit *A*_*i*_(λ) are calculated by rewriting eq 1 as a matrix equation. The best-fit value for *A* given populations *X* and a data set Δ*A* (also a matrix) can be calculated by approximating *A* ≈ Δ*AX* ^–1^ using the Moore-Penrose matrix pseudoinverse. We note that there is no exact inverse unless the model exactly reconstructs the data. The procedure for calculating the predicted spectra for a choice of the parameters θ is as follows: use θ to calculate *X*_*i*_(*t*), find the best-fit value for *A*_*i*_(λ) given *X*_*i*_(*t*), and get ψ_θ_(λ,*t*) = ∑_*i*=1_^*N*^ *A*_*i*_(λ)*X*_*i*_(*t*) (see eq 1).

For this work, we will take the perspective that the linear parameters are determined once *X*_*i*_(*t*) is calculated. In other words, we will treat the model as depending only on the nonlinear parameters. From a Bayesian perspective, this is equivalent to treating *A*_*i*_(λ) as “nuisance parameters”;^29^ for any choice of θ, the best-fit value of *A*_*i*_(λ) is used throughout the fitting and analysis. We then proceed to minimize the model error given by the chi-squared function^17,30^

4 $$\chi^{2}\left(\theta \right)=\sum_{i,j}\frac{{\left(\psi_{\theta }\left(\lambda_{i},t_{j}\right)-\Delta A\left(\lambda_{i},t_{j}\right)\right)}^{2}}{\sigma_{\mathrm{ij}}^{2}}$$

Note that as the kinetic parameters θ change, the spectral parameters are updated to the best-fit values for that choice of θ. As demonstrated in prior works,^18,20^ this error analysis can be performed using a standard nonlinear least-squares fitting algorithm. Details on the fitting procedure, including parameter initialization, can be found in the Materials and Methods Section below as well as Section S1 in the SI. The model description and fitting procedure described above have been widely used, but lack a thorough calculation of parameter uncertainty. Next, we extend the procedure to numerically estimate parameter uncertainties, which is highly desirable in the field from parameter evaluation, model refinement to a deepened mechanistic understanding.^4,5,31^

### Probability of Parameters

For a given model and data set, various choices of parameters are more or less likely. For example, if a choice of parameters does not fit the data within measurement error, it is unlikely to be the true set of parameters that describe the system. Here we try to quantify this scenario in order to estimate the probabilities of model parameters when fitting a data set. From these probabilities we can then calculate uncertainties and confidence intervals.

We use a Bayesian framework to quantify the probabilities. Mathematically we are interested in *P*(θ|*D*), the probability of the parameters θ given a data set *D*. Using Bayes’ theorem, we can write this expression as

5 $$P\left(\theta |D\right)=\frac{P\left(D|\theta \right)P\left(\theta \right)}{P\left(D\right)}\propto P\left(D|\theta \right)\pi \left(\theta \right)$$

Since we are interested in comparing θ for a fixed data set *D*, *P*(*D*) is some (unknown) constant.^13,29,32^ If we assume the data and the model differ by some Gaussian noise, then $P\left(D|\theta \right)\propto e^{-\left(1/2\right)\chi^{2}\left(\theta \right)}$ as was shown previously.^21,33^ There is some subtlety in how to best deal with the spectral parameters to arrive at eq 5: It would be computationally impractical to include them all in the MCMC sampling, and trying to deal with them analytically results in a nonphysical model (see Section S1.4 in the SI for details). We treat them, as is implicitly done in a recent study,^7^ by fixing them at their best-fit values.

The prior distribution reflects some knowledge about the parameters, theoretically before the data set is collected. For example, if the rate for a process is known from a separate measurement this can be included here. Using a uniform prior (π­(θ) = constant) or uniform in a large, bounded region constrains the data the least. We use uniform priors in this work and choose large enough bounds so there is no effect on the result (unless the parameters are otherwise unbounded).

Using the form for normally distributed noise the probability follows $P\left(\theta |D\right)\propto e^{-\left(1/2\right)\chi^{2}\left(\theta \right)}\pi \left(\theta \right)$ . Assuming a uniform (constant) prior, this problem is exactly analogous to a thermal distribution of some variable *P*(*x*) ∝ e^–β*E*(*x*)^. The best-fit parameter is the one that minimizes the “energy” χ^2^(θ), but other low-energy choices of parameters are also sampled at a finite temperature. The amount of variation in the parameters that is accessible by the “thermal” sampling determines the parameter uncertainty. Importantly, the interpretation of the parameter uncertainty relies on the model being a good fit for the data. It is possible to have a very precise estimate for a parameter in a model that does not fit the observed data. For example, consider fitting a parabola with a line. There is a single best fit that minimizes deviation; however, it is woefully inadequate to represent the true parabola. This analysis can calculate how the variation in parameters affects the fit quality relative to the best fit. To assess the suitability of a model, the goodness-of-fit statistics and analysis of residuals must be done, which is outside the scope of this work. More details can be found in literature, and the simplest method is to calculate the reduced chi-squared statistic χ_ν_^2^(θ) = χ^2^/(*N*_samples_ – *N*_vars_).^17,30^ If the residuals are only due to noise, χ_ν_^2^ ≈ 1.

### Markov Chain Monte Carlo (MCMC) Sampling

From the previous section, the probability distribution follows $P\left(\theta |D\right)=Ne^{-\left(1/2\right)\chi^{2}\left(\theta \right)}\pi \left(\theta \right)$ . The most straightforward way to estimate this, or any computationally difficult probability distribution is using MCMC sampling. Briefly, MCMC sampling works by iteratively undergoing a random walk in θ. Starting from some initial choice of θ_1_, a random step to a new set of parameters θ_2_ is proposed and the step is accepted probabilistically based on the ratio of *P*(θ_1_|*D*) and *P*(θ_2_|*D*). If the right acceptance rule is chosen (see Section S1.4 in the SI), the random walk will converge to the desired distribution *P*(θ|*D*). By doing this, normalization *N* is not needed.

There are a variety of technical challenges when implementing MCMC sampling efficiently. Fortunately, this technique is widely used and many of these challenges have been addressed in the existing open-source software. For this work we used the “stretch-move” method developed and implemented in the Python package “emcee”.^34^ The “stretch-move” was also used by Ashner et al.^7^ More details on the implementation are discussed in the Materials and MethodsSection and SI. It is interesting to note that this method was also proposed decades ago, but not implemented due to the limited computational power available at that time.^17^ This is no longer the case. The analysis for this work was performed on a laptop computer and generally took a few minutes to run the full analysis protocol on rather simple models like catechol and 8-methoxy-1,3,6-pyrenetrisulfonate or MPTS (see below). Our data sets generally consisted of ∼100 time points and ∼1000 wavelengths. It is worth mentioning that the computational cost of the χ^2^(θ) function scales linearly in the number of wavelengths, number of time points, and number of kinetic parameters, which would affect the ease of analyzing larger (or smaller) data sets. As a practical example, the most complex model for phiYFP as a fluorescent protein biosensor with 12 kinetic parameters and full analysis took around half an hour even on a laptop computer, obtaining good histograms as shown below in the Section “Applying to Real Experimental Datasets”. Future implementations of data uncertainty analysis can definitely be much faster; for example, the MCMC sampling can be sped up using parallelization (*e.g*., using multiple CPU cores) such as that included as a feature of the “emcee” package in Python.^34^

### Summary of Analysis Procedure

Before analyzing any data sets, here is a brief summary of the analysis procedure:

1. Choose a kinetic model to describe the data set.
2. Implement a nonlinear least-squares optimization algorithm, and find the best-fit kinetic parameters θ for the data set. This can be used to generate the population dynamics *X*_*i*_(*t*) and the spectra *A*_*i*_(λ) for each species.
3. From the best-fit parameters, use MCMC sampling to estimate the probability distribution for the parameters *P*(θ), which is used to generate uncertainties.

### Applying to Generated Data Sets

To assess the ability of the MCMC sampling to estimate *P*(θ), we generated transient absorption data at a single wavelength containing contributions from two species decaying in parallel. The χ^2^(θ) surface can be quite complex for even fitting just a biexponential decay, a fact which was previously reported and makes parameter estimation difficult.^14,15^ The two species had lifetimes chosen arbitrarily as 1.5 and 4.6 ps (inspired by the experimental example on anthracene and cyanoanthracene and recent examples on various green fluorescent protein chromophore derivatives).^4,35^ The species were excited using laser pulses centered at time zero (*t* = 0) with an FWHM of 100 fs, although this had little effect due to the relatively long decay lifetimes. Finally, noise was added to create a data set with a signal-to-noise ratio (SNR) of 100 and one with an SNR of 20, both of which are in realistic experimental ranges.^14,26^ For example, the former SNR would be considered good data quality for a typical excited-state absorption peak of ∼10 mOD (10^–2^ OD) with a typical noise level at 100 × 10^–6^ = 10^–4^ OD using our experimental setup.^24,36^ For each of the data sets, the χ^2^ surface was calculated and MCMC sampling was performed starting from the kinetic parameters used to generate the data.

Results for the MCMC sampling and χ^2^ surface calculation are shown for an SNR of 100 (Figure 1b) and 20 (Figure 1d), and the generated data used for fitting are shown as inset plots (Figure 1a,c). Note that the reduced χ^2^ was plotted to better compare to the calculation.^14^ To remove degeneracy in the model, the lifetimes were restricted so that τ_1_ ≤ τ_2_, and the off-limits region is indicated by the semitransparent white shading. In both cases, the χ^2^ shows a complex dependence on both parameters. The best-fit parameters are located in a curved minimum within a long “valley” that extends toward τ_1_ → 0 (the left) and τ_2_ → ∞ (the top). Intuitively, these limits can be associated with a model involving only one parameter. As τ_1_ → 0, the only population present is species 2. As τ_2_ → ∞, the population of species 2 is constant and the dynamics are entirely due to species 1. This phenomenon occurs in a variety of models that depend nonlinearly on the parameters, and can be used for model simplification.^37^ Outside of this valley the χ^2^ increases rapidly, indicating a bad fit to the data.

**Figure 1:**
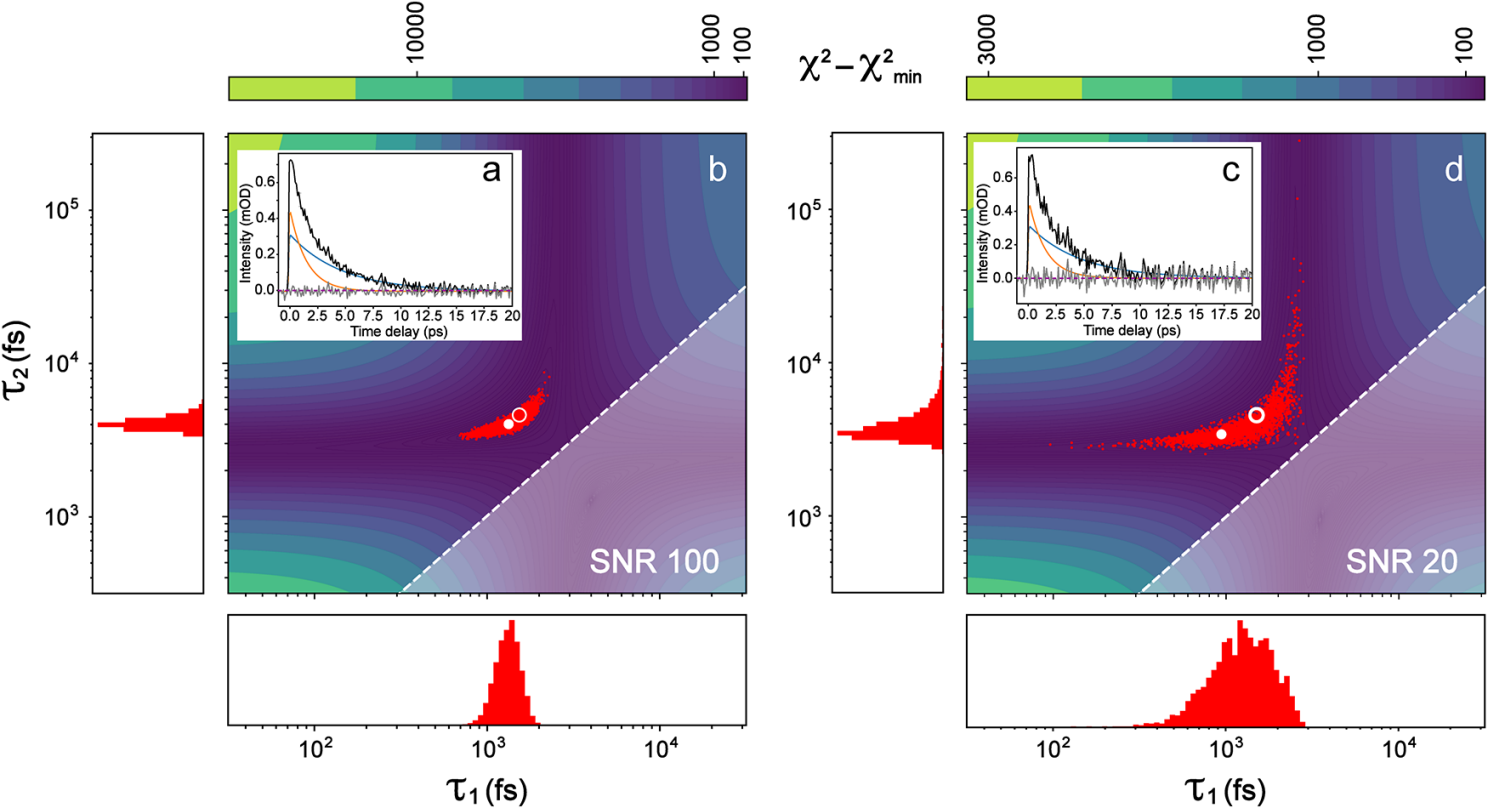
Error analysis for a generated data set. Curves (a, c) for a biexponential decay with lifetimes of 1.5 and 4.6 ps analyzed for a single wavelength, along with χ^2^ surfaces (b, d) as a function of the lifetimes (here keeping *t*_0_, FWHM fixed). To remove degeneracy, lifetimes are constrained so that τ_1_ ≤ τ_2_, *i.e*., the shaded triangle region in (b, d) is inaccessible. The white solid dots indicate best-fit parameters while the white hollow circles denote true parameters. The χ^2^ surfaces show a clear nonlinear dependence on lifetimes, though with an SNR of 100 (a, b) the parameter estimates are still highly constrained. However, with an SNR of 20 (c, d) the parameter estimates become skewed and much broader (see the side panels by 2D-contour plots). The skew in panel (d) can be attributed to a lifetime going to 0 (toward the left) or infinity (toward the top).

The effect of noise on the χ^2^ can be seen most clearly in the surface scale. Higher noise reduces the χ^2^ increase from deviations in fitting the data (see eq 4), nicely confirmed by the much reduced intensity in Figure 1d versus [Fig fig1]b. The probability distribution of the parameters, estimated from the MCMC samples, changes significantly. With an SNR of 100 (Figure 1b) the probability distribution is elliptical but mostly contained in a narrow region, allowing the relatively precise determination of underlying parameters. The elliptical shape indicates covariance in the parameters, which is expected in most nonlinear modeling. When the amount of noise increases (Figure 1d) the parameter distribution starts to extend along the valleys. On the χ^2^ surface this is still a relatively small region of parameter space, but the probability distributions of lifetimes are individually broad and asymmetrical (see side panels of Figure 1d). The shape of the χ^2^ surface causes the probability distribution to broaden rapidly, which is why exponential fitting is considered relatively difficult: a small increase in noise can create a huge region of “good fits” in parameter space, as corroborated by the larger separation between the best-fit parameters and true parameters (Figure 1d) than that with a higher SNR (Figure 1b). This finding also emphasizes the need for experimentalists to properly collect data and achieve the highest SNR allowed by the setup for each experiment on the molecular system of interest.

Another feature of fitting exponential decays is the difficulty in estimating lifetimes that are similar to each other. Previous work has found that in the presence of noise, the optimal fit for two lifetimes becomes a single-lifetime model.^38^ To examine this effect using our current workflow, we calculate the probability distributions of parameters for a generated biexponential decay with the fixed noise but increasingly similar lifetimes (Figure 2), generated with identical IRF parameters as Figure 1. In this case, the noise was generated with a standard deviation of 0.03 and corresponds to an SNR between 30 and 90 for all data sets (Figure 2a–d). For each of the data sets, the χ^2^ surface and parameter distributions were calculated as those in Figure 1.

**Figure 2:**
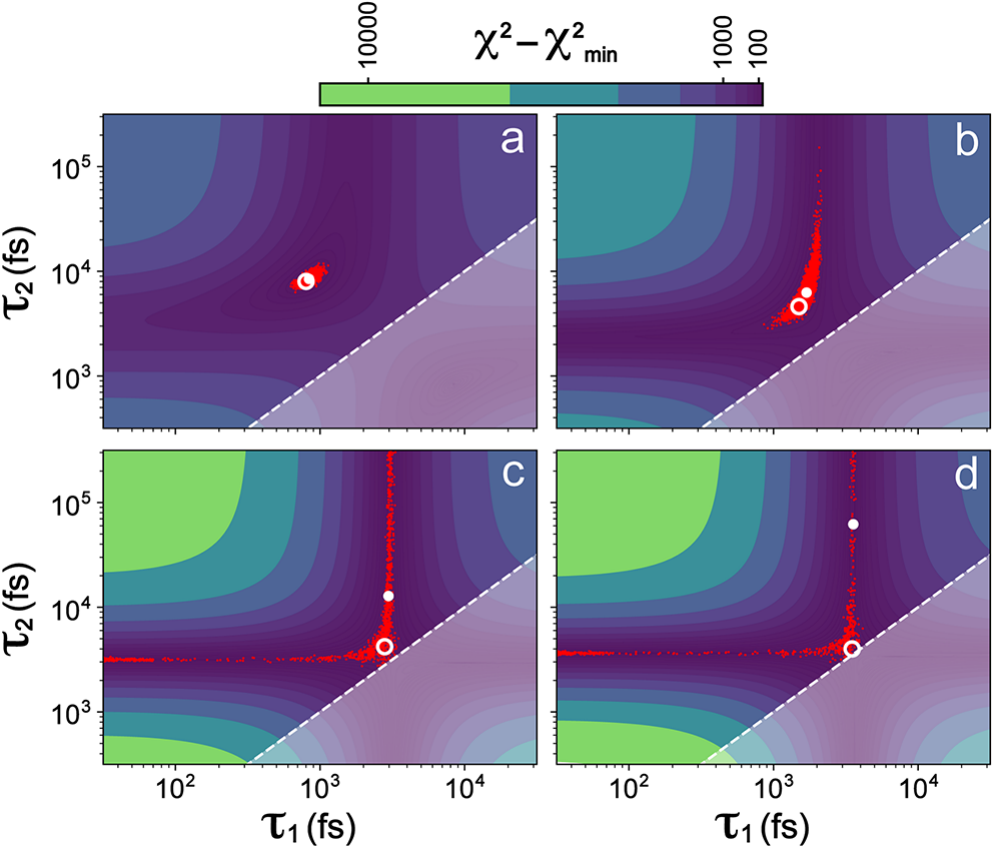
χ^2^ surfaces as a function of the lifetimes (with IRF parameters fixed) when fitting biexponential decays with added noise as the lifetimes become increasingly similar. The fixed amount of noise with σ = 0.03 was added to all curves. The pairs of lifetimes used to generate the data were (a) 0.8 and 8 ps, (b) 1.5 and 4.6 ps, (c) 2.8 and 4.2 ps, and (d) 3.5 and 4 ps. To remove degeneracy, lifetimes are constrained so that τ_1_ ≤ τ_2_, *i.e*., the shaded region is inaccessible. The white solid dots indicate best-fitting parameters while the white hollow circles denote true parameters; their differences grow larger with less distinguishable lifetimes (panels a–d). As the lifetimes approach each other (see panels going bottom right), the probability distribution of lifetimes becomes less constrained. In particular, when lifetimes are almost identical the distribution extends toward τ_1_ = 0 and τ_2_ = ∞, indicating effectively a one-component fit.

As in Figure 1, the χ^2^ surfaces are characterized by an elliptical minimum in a long “valley” of parameter choices that can approximate the data reasonably well (*i.e*., the χ^2^ values are not too large). As the lifetimes approach each other, the depth of the minimum relative to the rest of the valley decreases (from Figure 2a–2d). Correspondingly, the probability distributions of parameters become increasingly unconstrained, and go from slightly elliptical (Figure 2a) to clearly extending from τ_1_ = 0 and τ_2_ = ∞ (Figure 2d). Again, these patterns correspond to the limiting case of a one-component model, where one species relaxes to the ground state instantly (τ_1_ = 0) or remains constant (τ_2_ = ∞). The large region of parameters with similar fit qualities can also result in the best-fit parameters (from the MCMC sampling) being very different from the true parameters (particularly obvious in Figure 2c,d). Small effects from noise or numerical uncertainties can be sufficient to bias the fit to some other location in the valley of minima.

Experimentally, this means that determining whether a kinetic feature is due to a single transition, or multiple similar transitions becomes incredibly difficult. This same principle would apply for a data set generated with a distribution of similar parameters. In practice, this means that if the estimated error bars for a pair (or set) of lifetimes overlap and go toward ∞ or 0, the model could be fit just as well with only a single lifetime to capture the dynamics. Notably, this is true only for a parallel kinetic model wherein all the decay components are independent of each other.^11,12^ In a sequential model with identical (or nearly identical) rates the kinetics can no longer be described accurately as the sum of exponential decays, and so the model can no longer converge to a one-component fit. Instead, different cases can have very large uncertainties. For example, if there are two very different lifetimes with similar amplitude weights, the dynamics will be dominated by the longer one. However, we should stress that this is dependent on the model used to generate data, and less on the model used for fitting. The parameter uncertainty in fitting the sequential model assuming parallel decays looks almost identical to fitting the proposed sequential decays;^12,39^ the only difference is in the recovered amplitudes from the two species. Similarly, fitting a parallel model using sequential kinetics has the same properties as shown in Figure 2. These results uncover one benefit of this calculation: it can accurately identify when the data can be fit with a simpler model, which can be described more generally as the “manifold boundary approximation method”.^37^

Measuring a single curve to recover kinetic parameters is a nice test case for estimating parameter uncertainty and recognizing its intrinsic limitations, although it does not capture most real experiments. One of the key features of global analysis is its ability to measure the dynamics across multiple wavelengths and reach a “globally” accepted kinetic model representation. Previous work has shown that using multiple curves, at different wavelengths, effectively constrains the χ^2^ surface, allowing for a more accurate determination of model parameters.^14,15,35^ However, there has been no explicit calculation of the change in parameter uncertainty as a result. To verify this point and demonstrate the effectiveness of MCMC sampling in a more realistic context, we generated the multiwavelength transient absorption spectra and sampled them at various probe wavelengths. The dynamics are identical to those used in Figure 1, and the spectra for each species *A*_*i*_(λ) were chosen to be Gaussians with differing center wavelengths, widths, and amplitudes. These spectra (*i.e*., artificially generated data sets) are shown in Figure 3a, while the set of curves used for fitting are shown in Figure 3b. The analysis was performed on each wavelength individually and on the entire set to determine the effect of implementing “global” analysis.

**Figure 3:**
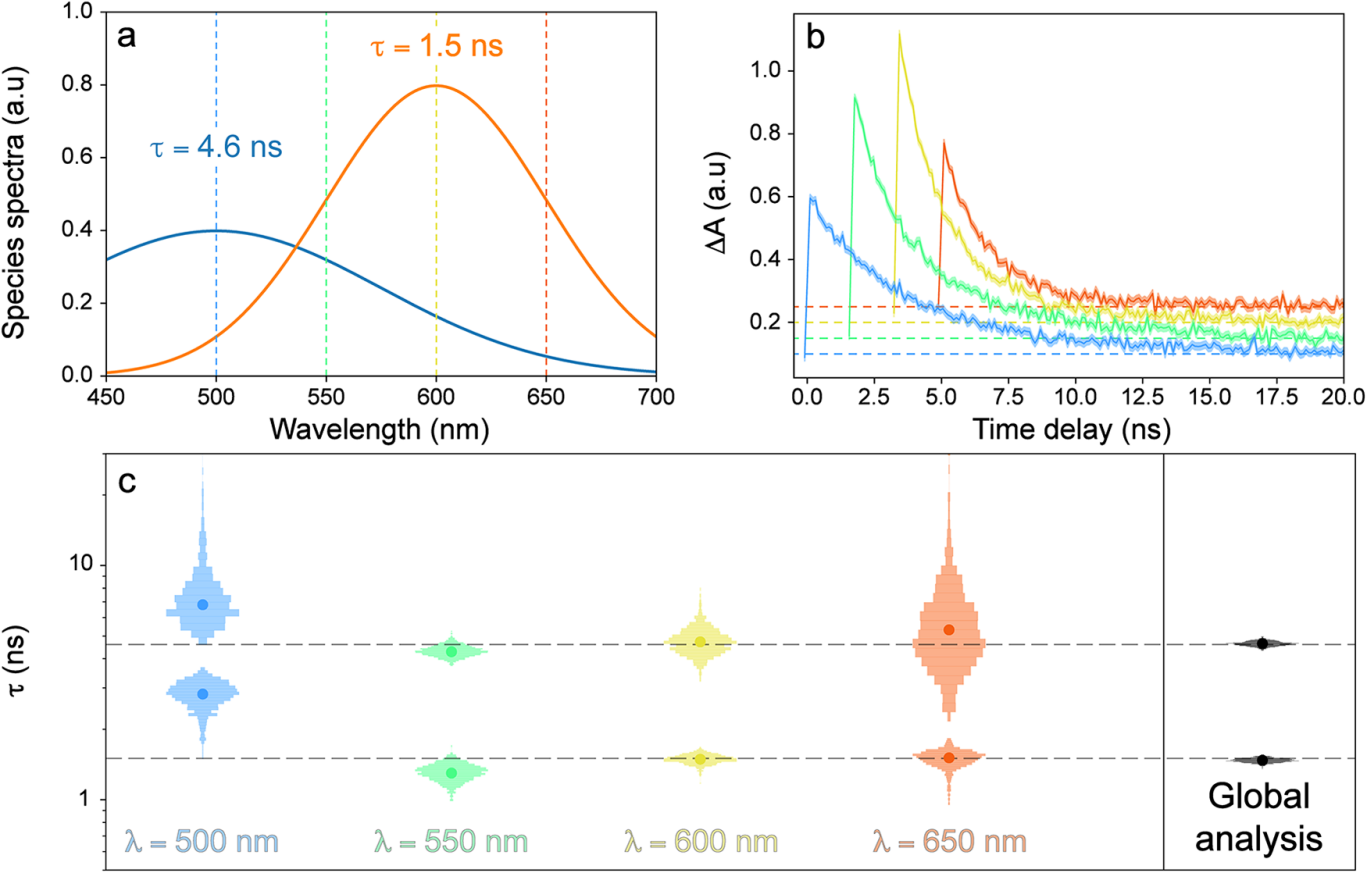
Parameter determination using probe-dependent and global analyses. (a) Two species with relatively similar lifetimes (color-coded) but distinct absorption peaks. (b) Upon simultaneous excitation, the two species would decay at different rates as tracked by four probe-dependent dynamic traces (color-coded). (c) The dynamics at four probe wavelengths (labeled at the bottom) were measured to estimate the two lifetimes, while their true values are denoted by horizontal dashed lines. The best-fit lifetimes are indicated by solid dots, and the estimated probability distributions are shown using vertical histograms. Global analysis results (with the clearly diminished uncertainty) are shown to the right side of the vertical black line.

The results of the parameter estimation (Figure 3c) show the best fit and probability distributions for the two lifetimes from each of the analyses. The best-fit kinetic parameters recovered vary significantly between the wavelengths, which matches what is experimentally observed when fitting single-wavelength regions: the kinetic parameters vary quite a bit between spectral regions, even if the underlying kinetics are thought to be the same. These results, which match the findings from Knutson et al.,^35^ suggest that this could be due to a high uncertainty in the parameter estimates rather than a true difference in kinetics—a known problem for using the probe-dependent fit alone to interpret dynamics, especially for the overlapped spectral features undergoing varied dynamic changes.^5,11,40^ This useful point can be corroborated by probability distributions, also shown in Figure 3c. Despite the clear range in best-fit estimates (solid points), the probability distributions of parameters for all wavelengths have significant overlap. The effect of the relative spectral amplitudes of two species can also be seen. The bluest (reddest) region has the least signal from the 1.5 ns (4.6 ns) lifetime component (see Figure 3a), and correspondingly has a very large uncertainty in that parameter (Figure 3c).

Fitting all wavelengths simultaneously, using global analysis, has the much better accuracy and precision than analyzing all the wavelengths individually (Figure 3c). The recovered best-fit parameters are almost exactly the parameters that generated the data set, and the parameter uncertainty is quite narrow. There are two intuitive explanations for this effect. First, there are simply more data points so the noise can be averaged out. This effect is not insignificant, especially in analyzing real experimental data where thousands of wavelengths can be measured simultaneously in the pump–probe setup. Second, global analysis uses regions with different ratios of amplitudes from each species and finds the kinetic parameters that are consistent across all the measured wavelengths. This intrinsic mechanism suggests that global analysis can also mitigate the effects shown in Figures 1 and 2, thereby getting rid of the long “valleys” that cause large parameter uncertainty.

In addition to the inherent difficulties in fitting exponential dynamics, there can be cases where the system dynamics do not suit the experimental time scale. Most commonly the dynamics could be much faster than the time resolution (either due to IRF or the measured time points) or are much longer than the measurement time window. In practice there is no exact rule to determine whether the kinetic parameters can be estimated, since it depends on the specific data set (*e.g*., how much noise, what the dynamics of the other species are). Using MCMC sampling to estimate parameter uncertainties can determine this desirable property quantitatively for a given data set and model. For simplicity, we demonstrate this point using a generated transient absorption data set for a single species whose lifetime is longer than the measurement duration. Specifically, we vary the lifetime from 2 to 50 ns (Figure 4a–c) with a measurement duration of 1 ns, roughly corresponding to a motorized translation stage with 15 cm travel length (which is typical for ultrafast spectroscopy laboratories).^36,41,42^ For each of the data sets, we vary the assumed uncertainty level, and calculate the probability distributions for the lifetime from fitting. In addition, the χ^2^ surfaces are shown to compare directly with the histogram. Note that no noise is actually added to the data set to make comparing the histograms between noise levels easier. The shape of χ^2^ surface changes with noise, resulting in stretched probability distributions for the higher-noise cases. The χ^2^ surface was computed assuming a noise of 1 (σ = 1 for the uncertainty), *i.e*., plotting $\epsilon \left(\theta \right)=\frac{1}{2}\sum_{i,j}{\left(\psi_{\theta }\left(\lambda_{i},t_{j}\right)-\Delta A\left(\lambda_{i},t_{j}\right)\right)}^{2}$ . Note that in this case, we plotted the probability as $e^{-\left(1/2\right)\chi^{2}\left(\theta \right)}=e^{-\epsilon \left(\theta \right)}$ with the expression for χ^2^(θ) shown in eq 4 (*i.e*., the model error divided by the uncertainty), which was used for error analysis and generating the histograms. The MCMC samples for the two choices of σ were rescaled to match the curve (see eq 4).

**Figure 4:**
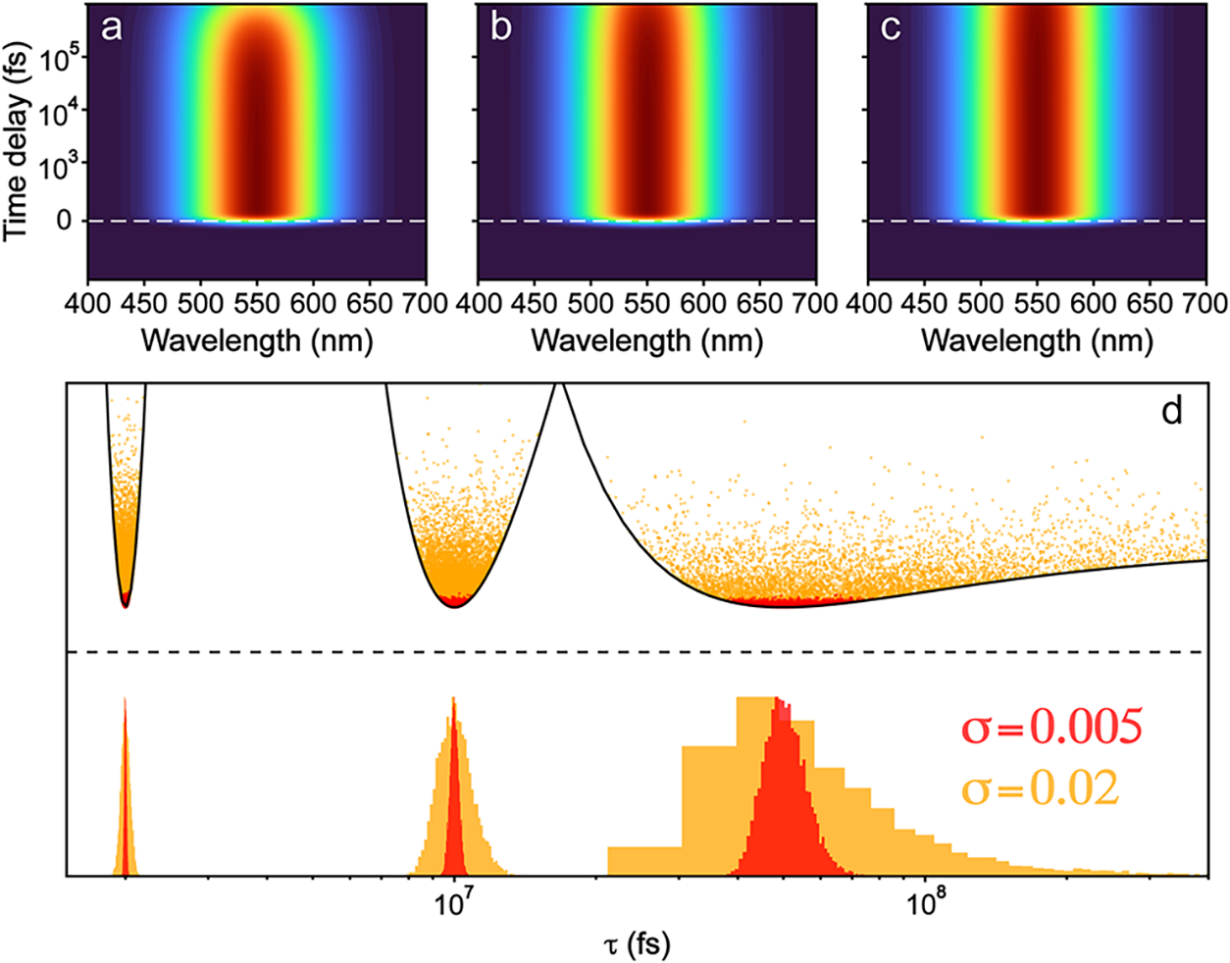
Generated transient absorption spectra for a single species, with increasing lifetimes of (a) 2 ns, (b) 10 ns, and (c) 50 ns. No noise was added. (d) The χ^2^ surfaces (top) are shown for each lifetime case, as well as the histograms (bottom) and points from MCMC sampling (top), assuming two levels of noise (red and orange).

As the lifetime increases, the minimum of χ^2^ surface becomes much broader and asymmetrical (Figure 4d). This fact is independent of the noise level (and would exist with added noise). The breadth reflects the fact that the change in fit quality becomes increasingly insensitive to the lifetime used for fitting as the decay becomes slower. For a 50 ns decay, the signal would only decay around 1 – e^–1/50^ ≈ 2% over the measurement. Using a lifetime of 100 ns would lead to a difference of ∼1% in the signal. For the 2 ns lifetime the signal decays by ∼40%, and doubling the lifetime has a much larger effect. The asymmetry is due to the fact that decreasing the lifetime has a much larger effect on the fit quality. This results in the ϵ­(θ) surface becoming flatter as it extends toward longer lifetimes. Such an analysis provides some credence for a common practice in the field by giving a lower bound for the longest lifetime which could be somewhat limited by the detection time window, also having the largest uncertainty for that specific parameter (Figure 4d).

The effect on the parameter estimate depends on the noise present in the data, as can be seen by the varying shapes of the histograms in Figure 4d. For a physical intuition, we can imagine the two noise levels as ensembles at different temperatures sampling the same potential (given by the ϵ­(θ) surface). This analogy is mathematically accurate, since the samples follow *P*(θ) ∝ e^–ϵ(θ)/σ^2^^ (from eq 4), and clearly σ^2^ plays the role of temperature (here the noise is uniform). For higher levels of noise, the ensemble of samples can access points higher on the ϵ­(θ) surface and therefore a broader distribution. For the 50 ns lifetime, this causes the distribution to become highly skewed toward longer lifetimes and very broad. One interesting phenomenon to note is that of “parameter evaporation,” which occurs when the noise is high enough for the ensemble of samples to leave the minimum and extend toward longer lifetimes (*e.g*., see the 50 ns lifetime case in Figure 4d). Since the surface becomes flat, the distribution is free to extend toward infinity (or the upper bound determined by the prior) and the parameter becomes effectively completely unconstrained.^13^ This can be thought of as evaporation since it is analogous to a particle escaping an interaction potential at some finite temperature; even though there is still an energetic preference for being bound, being unbound is favored entropically if the temperature is large enough. In other words, even though there is a higher likelihood of being near the best-fit parameter value, there is a huge region of similar probability extending toward infinity.

### Applying to Real Experimental Data Sets

Finally, we apply this method to some experimental data sets to assess the parameter uncertainty estimated under real conditions. Since our current Python code did not capture any chirp or coherent artifacts, we focused on data sets that had relatively simple kinetics and minimal IRF features that needed to be fit. Interpreting the parameter uncertainty as reflecting the physical dynamics relies on the model describing the data accurately. Future work could easily extend this framework to include more complex IRF functions, covering the effects of oscillations,^22^ since they would appear in the kinetic parameters θ. Qualitatively, we expect that they would not have a large effect, except for likely increasing the uncertainty in the fastest kinetic parameters (*e.g*., the femtosecond-scale IRF effects would have little impact on the nanosecond decays).^4,5^ More parameters usually allow more variance, since there are more degrees of freedom.

Measurements of catechol in acidic solution fit with a three-component sequential model are shown (Figure 5) with slices at various probe wavelengths taken from global fits (Figure 5a), the evolution-associated difference spectra (EADS, Figure 5b) on the basis of a sequential kinetic scheme,^12,24^ and the probability distributions associated with each kinetic parameter (Figure 5c-g) calculated by MCMC sampling. The mean and confidence interval were calculated from the MCMC samples, using two standard deviations. A three-component fit was chosen as the minimal number required to fit the observed spectral dynamics, which well match the kinetic modeling on a similar system in aqueous solution at pH 7.4 (versus our sample at pH 4).^43^ There is clear difference between the excited-state absorption (ESA) band shifted to the long-wavelength side (above ∼500 nm with a peak around 580 nm, see Figure 5b) for catechol in acidic water and a broad ESA band from ∼350 to 700 nm for catechol in slightly basic water, yet the underlying intramolecular charge transfer, solvation, and internal conversion dynamics observed^43^ are similar up to ∼1 ns as collected using our experimental setup (Figure 5a).^44^

**Figure 5:**
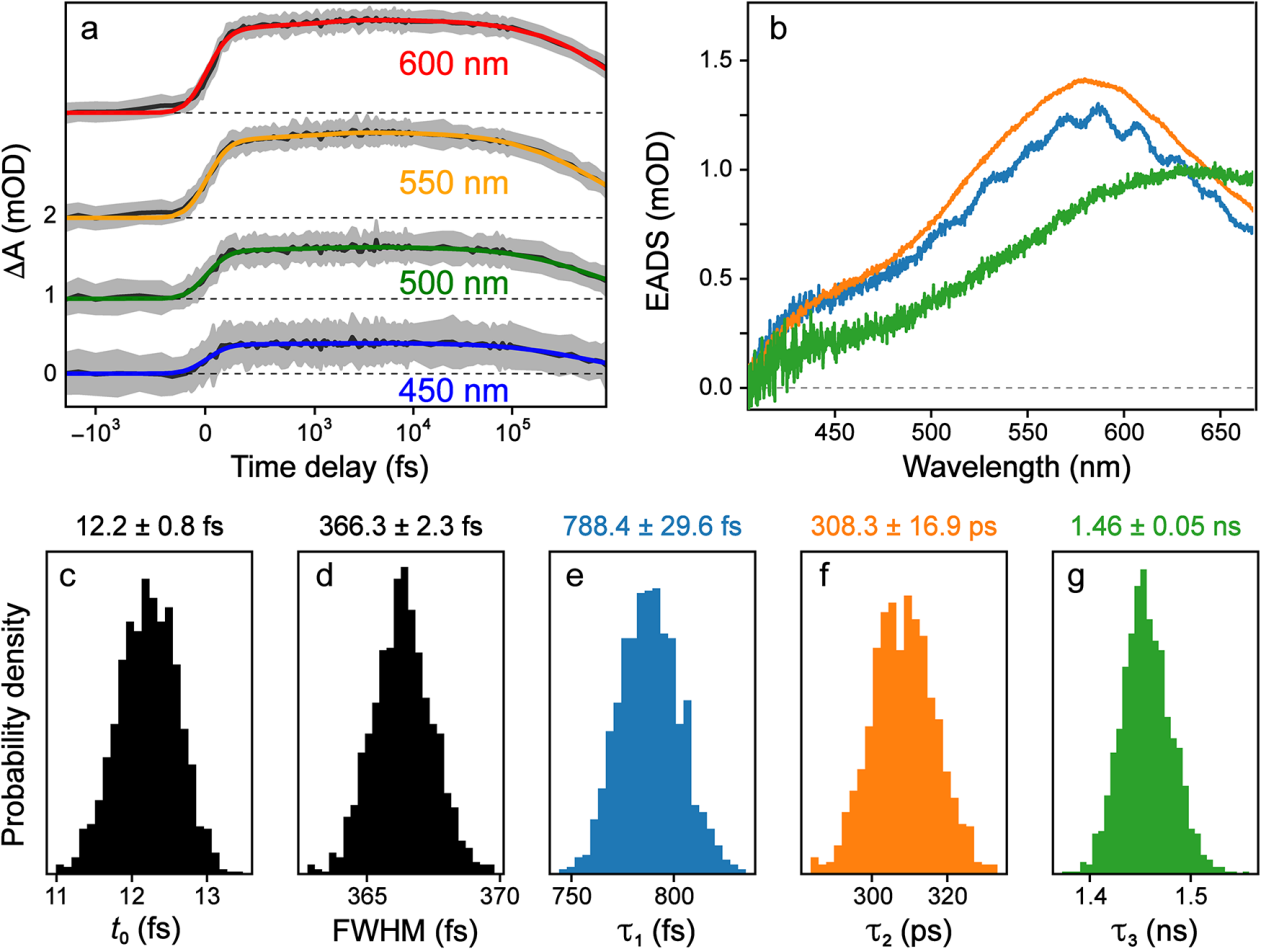
Global analysis of the transient absorption spectra of catechol in acidic (pH 4) buffer after 267 nm excitation, fit with three species in a sequential model. Fits from global analysis are shown (a) at several characteristic probe wavelengths denoted in the insets (the color-coded solid traces denote the global fits achieved across the spectral range while the gray shade shows the noise level), and with (b) the resulting evolution-associated difference spectra (EADS, color-coded) for each species. (c–g) The probability density distributions of kinetic parameters are shown as histograms, calculated from MCMC sampling.

Using a parallel model, the only difference is in the retrieved spectra (decay-associated difference spectra, DADS)^12^ versus EADS (see above) as expected.^39^ In this case, all the kinetic parameters were calculated to exhibit well-defined probability distributions, and the relative uncertainties of all parameters are on the order of a few percent. The duration and line shape of the model components suggest that they are not fitting coherent artifacts, but instead physically meaningful features from the excited-state species (apart from small oscillations in the first component, see the blue trace in Figure 5b). There are some minor discrepancies in the model at early time points, which indicates that some additional features need to be added to the model to fully describe the observed spectral behavior. However, this difference is only on the tens of fs time scale which would have little effect on the retrieved underlying components and “functional” dynamics of interest for the molecular system. From our analysis, each retrieved kinetic component is well separated with highly normal probability distributions (Figure 5c–g). Based on our aforementioned testing with various computed data sets (Figures 1–4), these lifetime parameters appear to be the well-confined minima within the χ^2^ surface, giving high confidence in the global fit method.

We also applied this analysis to a previously analyzed transient absorption data set, MPTS in water upon 400 nm excitation. Using a typical three-component model for organic chromophores in solution on ultrafast time scales,^45^ we were able to accurately fit all dynamic features (Figure 6a) and our custom-made Python code recovered almost identical results as those from global analysis using the Glotaran software (Figure 6b).^12^ The uncertainties in kinetic parameters are very small due to the large SNR in this data set, as highlighted by uncertainty levels in Figure 6a (with SNR ≈ 1000) versus Figure 5a (with SNR ≈ 10). While the recovered components and lifetimes are very similar to the results from global fits, the actual numerical values are not within error. In particular, the longest lifetime retrieved for MPTS in water (3.1 ns in Figure 6g, associated with the fluorescence state lifetime) is notably shorter than that from global analysis (3.8 ns)^45^ using the open-source software Glotaran.^12^

**Figure 6:**
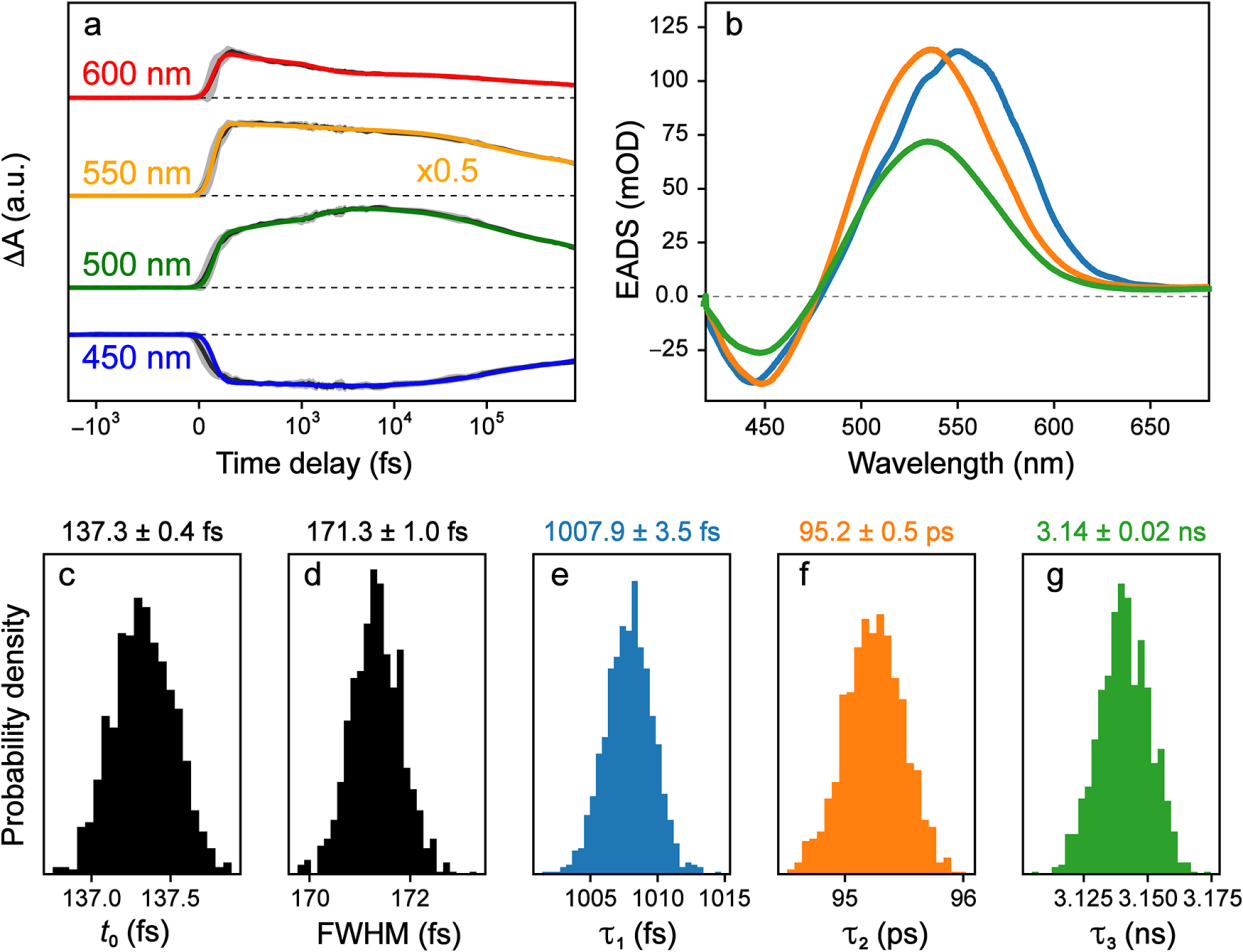
Global analysis of the transient absorption spectra of MPTS in water after 400 nm excitation, fit with three species in a sequential model. Fits from global analysis are shown (a) at several characteristic probe wavelengths denoted in the insets, and with (b) the resulting evolution-associated difference spectra (EADS, color-coded) for each species. (c–g) The probability distributions of kinetic parameters are shown as histograms, calculated from MCMC sampling.

One possibility is that Glotaran is able to capture a shifted time zero, while the model used in this work with custom Python code does not. This difference likely would exert an effect, particularly on the earlier dynamics; however, it is hard to imagine it would have such a large effect on a ns-scale decay (*i.e*., with a significant separation of time scales). As a demonstration, manually offsetting *t*_0_ to 450 fs (a huge change from the best-fit value of ∼140 fs) results in the longest lifetime changing only from 3.14 ± 0.02 ns to 3.02 ± 0.02 ns (the analysis can be found in the code on GitHub, see the “Data Availability” Section below for the link). It is worth noting that the lifetime of 3.1 ns for the final component is much more consistent with the probe-dependent fits shown for multiple probe wavelengths, and for the same fitting performed on the transient excited-state Raman data.^45^ Finally, it is possible that the noise is not totally decorrelated between wavelengths and/or time points; this would overestimate the amount of data, and result in over confinement of some parameters.

An in-depth analysis of noise is outside the scope of this work, but we briefly investigated this issue by repeating the fitting and subsampling the wavelengths (*e.g*., using every 10 or 50 wavelengths instead of using all the available wavelengths, see Figure S4 in the SI). This approach resulted in an increase in error bars but no significant change in the best-fit estimates of kinetic parameters. We also performed the error analysis using a much reduced signal-to-noise ratio for catechol in acidic (pH 4) buffer after 267 nm excitation (Figure S5 in the SI) for a direct comparison to the analysis results in Figure 5, obtaining larger error bars (particularly for certain long time constants lacking the sufficient data support) but still validating the robustness of MCMC framework. A full model, including a more realistic IRF, could better resolve these discrepancies in the future, but our current analysis demonstrates the ability to quickly calculate probability distributions and provide valuable feedback on quality when analyzing complex spectral data sets.

Last but not least, we investigate the uncertainties in a much more complex model using target analysis. The standalone excitation-ratiometric yellow fluorescent protein chloride sensor (phiYFP) was found to have a bifurcated relaxation pathway in the presence of chloride ions, and we fit the data using the same kinetic model depicted in Figure 7a.^46^ The kinetic matrix we used is shown in the SI (Section S1.4.3). Because this process has fast initial kinetics and strong coherent artifacts, we used three initial components with very short lifetimes in parallel (one of which decayed into the rest of the kinetic model)^46^ in order to capture the signal near time zero. With a more complete description of the IRF, this step should not be necessary.

**Figure 7:**
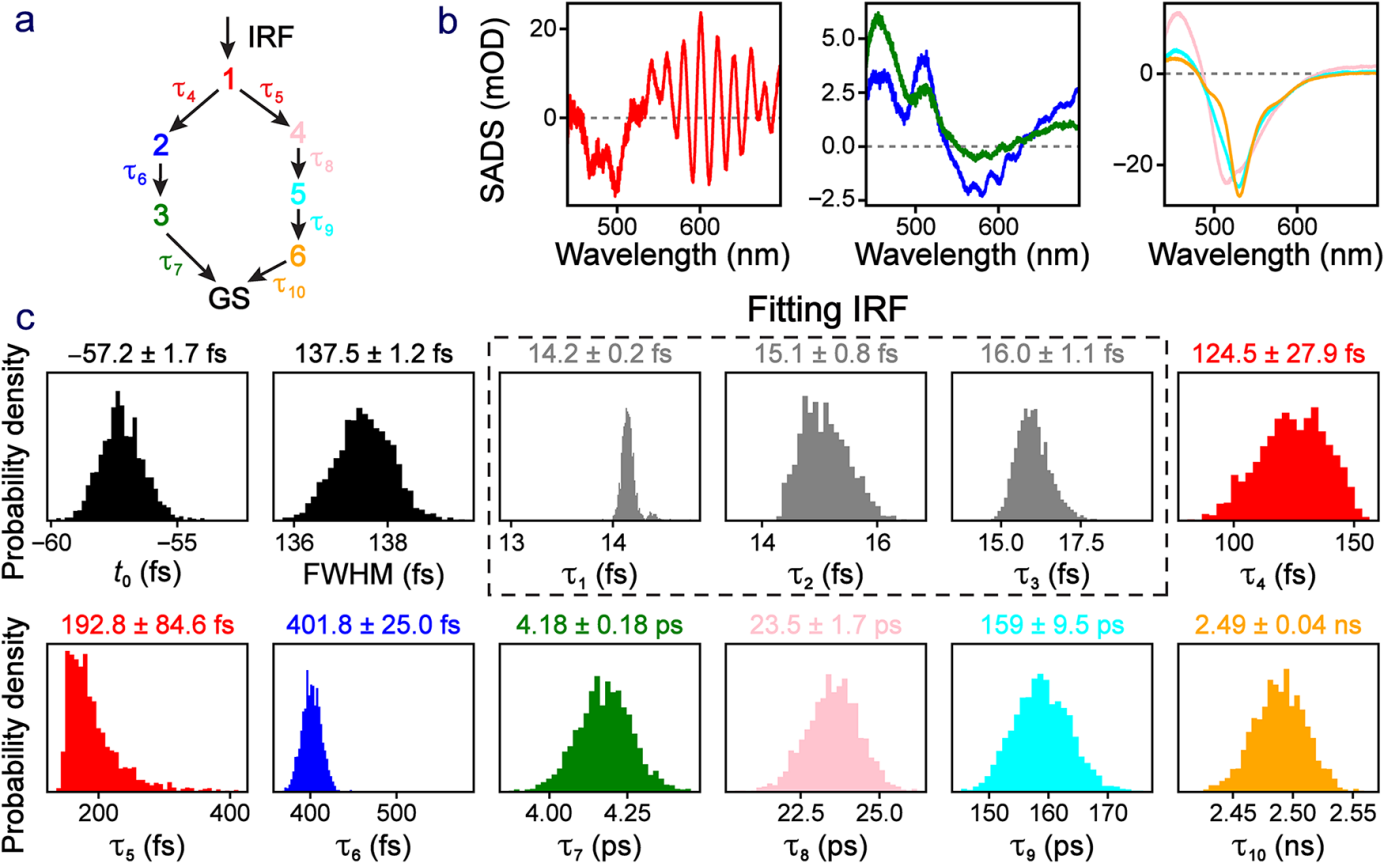
Global analysis of phiYFP in aqueous buffer solution. (a) The kinetic model for the chloride sensor working mechanism with a protonated chromophore. (b) Fitting results in the recovered species spectra for the initially excited populations. (c) Probability density distributions for the retrieved kinetic parameters. The first three ultrafast lifetimes were used to fit experimental IRF features that were not parametrized in the IRF function, encircled by a dashed gray box. Specifically, in the model three species were initially excited: τ_1_ and τ_2_ are the decays of two components back to the ground state, and τ_3_ is the decay of a third component into state 1 (red). The other lifetimes (color-coded) reflect the kinetic scheme specified in panel a.

The recovered species-associated difference spectra (SADS, which could involve both parallel and sequential pathways in a proposed kinetic scheme)^12^ and probability distributions for kinetic parameters are shown in Figure 7 (upper right and bottom panels). The SADS correspond closely to our prior global analysis results with characteristic spectral profiles attributed to the ring-twisting (dim) and excited-state proton transfer (ESPT, bright) species,^46^ although we herein exclude the first state (associated with the Franck–Condon region) due to the IRF modeling deficiencies. We note that the retrieved probability density (error distribution) for the initial two bifurcated pathways out of the Franck–Condon region (τ_4_ and τ_5_ in Figure 7a,c) exhibit distinct patterns: a more symmetric Gaussian-like profile for τ_4_ (∼28 fs or 22% error) versus a more skewed profile for τ_5_ (∼85 fs or 44% error). This finding nicely reflects the nature of these two competing pathways: the major twisted intramolecular charge transfer (TICT) route (see the associated species spectra as blue and green traces in Figure 7b middle panel) and the minor ESPT route (see the associated species spectra as pink, cyan, and orange traces in Figure 7b right panel), which are also corroborated by the fluorescence quantum yield (FQY) of ∼6% upon 400 nm excitation of the protonated chromophore of phiYFP (*i.e*., confirming that the fluorescence-preceding ESPT reaction is indeed a minor pathway).^47^

The recovered kinetic parameters are overall a good match with the literature^46^ with some minor differences that could be attributed to the difference in IRF fitting, the same as in the MPTS data set (Figure 6). As in the catechol data set (see Figure 5 above and Figure S4 in the SI), if the number of wavelengths is reduced, the error bars for most of the parameters increase dramatically. For this more complex model on phiYFP the effect is more significant, while the simultaneous retrieval of seven lifetimes (τ_4_ to τ_10_) with characteristic dynamic profiles from key intermediate species substantiates the rigorous and accurate representation of molecular dynamics during the excitation-ratiometric chloride sensing process.

### Significance and Applicability of MCMC Sampling for Ultrafast Spectroscopic Data and Beyond

In this work, we demonstrate that MCMC sampling can easily estimate parameter uncertainty when appended to standard fitting methods used in existing software implementations. While outside the scope of this current work, it should be easy to add this functionality to existing software by incorporating an MCMC sampling step starting from the best-fit parameters. This would allow more widespread and easier reporting of parameter uncertainties with relatively little work for the software users and experimentalists alike. Furthermore, this method enhances researchers’ capability to deeply understand and convey complex chemical and material processes with improved confidence.

We should mention that one alternative approach that has been used in a few previous studies of parameter uncertainty was to scan the parameter space near the best fit.^15–17,30,48^ This method works well if the number of parameters is small and the region has a well-defined local minimum, although it would be difficult to implement for models with many parameters or complex χ^2^ surfaces. Another benefit of MCMC sampling can be a more accurate determination of best-fit parameters, compared to a standard nonlinear optimizer. Unlike a previous study,^7^ we did not use the MCMC sampling to find the best-fit parameters *ab initio*; we found that this is much less efficient than dedicated optimizers which use gradient-descent-based methods (like Levenberg–Marquardt, see Section S1.2 and Figure S1 in the SI). Often, especially for complex models or cases with large parameter uncertainties, the gradient-descent algorithm will end before finding the best-fit values. This seems to occur because the algorithm is near the minimum with a sufficiently low gradient. Running MCMC sampling can either confirm that the minimizer found the minimum, or will relax toward the true minimum. This would make fitting much more robust and accurate, especially in higher dimensions when nonlinear optimization can be difficult. In dedicated software packages, one could imagine a procedure where nonlinear optimization via Levenberg–Marquardt and MCMC sampling is repeated until results converge; we believe this would be incredibly robust and spare users from having to carefully adjust initial conditions or optimize parameters. In a cruder form and by hand, this was done in this work to validate our fitting methodology.

Outside of parameter estimation, adapting a probabilistic perspective on model fitting also provides nice opportunities for more careful modeling techniques. In this work we minimized the χ^2^, then connected it to the parameter likelihoods. Instead of the parameter probability, including a nontrivial prior that has information about the kinetic parameters or even species spectra, could be directly used during the fitting procedure. By choosing the prior to be a delta function, sharply peaked function, or an indicator function it can fix or constrain certain parameters, a practice which is already relatively common when fitting data sets. This could allow a convenient and unified way to fix, bound, or impose specific constraints on the model. For example, it could be possible to require the species-associated difference spectra (SADS, see above) to be non-negative or that certain rates must match separate experiments. It is simple to fix relationships between parameters (*e.g*., 1/τ_1_ + 1/τ_2_ = 1/*C*, to constrain the lifetime of a state with two relaxation pathways). The choice of the prior is also quite important in model comparison using Bayes factors.^32^ Previous work on a different model used the Bayes factor for model selection,^21^ and future work could further investigate the possibility of using it as an alternative and “user-free” method for effectively determining the number of species in global analysis.

Finally, this framework is much more connected to the rapidly growing machine-learning community and facilitates more cross-disciplinary (both theory and experiment) development.^49–53^ We would also like to stress that this perspective, or even the numerical technique of MCMC sampling, are not specific to global analysis and could be applied to other methods as well as vibrational data sets using femtosecond infrared and Raman techniques.^26,54^

Our results from applying this method to transient absorption data sets suggest that kinetic parameters generally have nontrivial covariance. In cases where the χ^2^ surface was explicitly calculated the minimum had elliptical shapes, even when the lifetimes were an order of magnitude different. This effect generally became stronger as the parameters become more similar. Despite this feature, parameters could still be well-constrained with a sufficient SNR. There are cases with extremely large parameter uncertainty, but this is usually due to a model pathology or measurement inadequacy (if the lifetime is too short or too long, relative to the measurement). Detecting and diagnosing these cases is a major application of MCMC sampling, which also suggests that a new model or modified experiment is required to obtain more accurate results for kinetics.

Notably, we find that global analysis is essential for accurate parameter estimation even in very simple models. As demonstrated in Figure 3, for a system with only two decays and relatively low noise, parameter estimates vary widely depending on the probe/detection wavelength chosen. The estimates tend to be worse as the spectra and lifetimes become less distinct. While the probe-dependent fitting can still be valuable,^27,45^ especially when trying to avoid nonphysical artifacts or to validate global models (cross-examination), it is likely more accurate to perform global analysis on a subset or all of the observed wavelengths rather than a fit on a single wavelength. When using global analysis, parameter uncertainty is quite low in experimental applications, and this is in large part due to the large number of wavelengths used in the measurement. This is what was observed by Ashner et al.,^7^ who used a relatively simple model to describe their data (with only three or four nonlinear parameters). Generally, we demonstrate that the parameter uncertainty is usually around a few percent, but can increase rapidly when a small number of wavelengths is used or when parameters are overlapping/similar like the bifurcating pathways for phiYFP (τ_4_ and τ_5_ in Figure 7a,c). Issues are expected to occur most often for short lifetimes that are overlapping or comparable to the IRF, long lifetimes that are longer or comparable to the measurement window, or in cases where the structure of the model has extra degrees of freedom.

There are many possible directions for continued research, particularly for selecting the number of species needed to fit a data set. If the number of components is more than required, the extra degrees of freedom should cause parameter uncertainty to increase. This was seen and used to justify the choice of model.^7^ However, in our case we found that adding extra kinetic components can fit the IRF artifacts or other small features of the system. For example, a continuous redshift can be fit through a series of components that no longer reflect distinct physical species.^55,56^ Imposing restrictions on the kinetic parameters or the spectra could prevent these issues and allow for a refined model selection.

Many real systems have features that cannot be easily captured by a simple global analysis model: inhomogeneous populations with a distribution of lifetimes,^57^ the spectrum from a given species can change continuously (*e.g*., solvation or vibrational relaxation causing redshifts),^4,5^ diffusion reaction dynamics for the bimolecular encounter,^58^ stretched exponentials for diffusional relaxation in noble-gas liquids or dynamic heterogeneity in proteins,^59,60^ and higher-order or power law kinetics are some examples.^61–63^ In these situations, the system does not satisfy the conditions of bilinearity, first-order kinetics, or a discrete set of species necessary for global analysis (as we have described it in this work). It is thus important to emphasize that uncertainty analysis of parameters is not a good way to check model validity, at least by itself. Instead the model validation can be conducted with carefully designed experiments, control samples, isolating certain variables, adjusting external conditions, etc. The uncertainty analysis procedure explores the cost surface near the best-fit parameters, but only relative to the best-fit parameters. The same principle applies if the number of components used is insufficient; the parameters would likely be very well constrained despite the model being a poor fit.

It is worth noting that some computational work suggests that distributions of lifetimes are almost indistinguishable from fitting with a discrete set, unless the distributions are very broad and the data have very high SNR.^57^ In other words, if it is unknown whether there should be inhomogeneous populations, comparing the results of either analysis will easily determine this. As well, spectral shifts can be fit by multiple components in a global analysis model; however, in this case the individual spectrum can no longer be assigned to distinct chemical species.^55,56^ Other methods exist but generally fall into “soft modeling” which generally lack parameters with clear physical interpretations, or “a priori” methods like direct fitting of peaks with Gaussians, which make many assumptions about the physical reality of the system. Global analysis tries to walk the line between these two extremes and provide important insights effectively and efficiently.

## Conclusion

In this work, we tackle precision chemistry from the error analysis perspective using computational methods. We proposed and demonstrated a robust method for calculating parameter uncertainty in global analysis that can be easily appended to the existing software, building on prior uncertainty analysis techniques. In short, after using standard nonlinear least-squares optimization, MCMC sampling can estimate the probability distribution. Many of the technical challenges can be solved using dedicated open-source software for this technique. This method can be generalized readily to more complex kinetic models or other parametrizations of optical artifacts, which can greatly aid the broad and vibrant community interested in time-resolved spectroscopy and molecular dynamics on ultrafast time scales to delineate myriad chemical reactions frame by frame.

Applying to generated and real data sets, we found that the method can robustly estimate parameter probability distributions, and detect cases where parameters are unconstrained. Often these cases occur in pathological cases, for example when two species have very similar kinetics or when a lifetime extends beyond the measurement duration: both scenarios were examined in this work. In experimental data sets across chemistry and biology disciplines, we find that the parameters can be very well-constrained, even in complex kinetic models. In particular, this finding depends strongly on using global analysis with many wavelengths measured simultaneously, instead of the probe-dependent fits at certain wavelengths, particularly when characterizing long lifetimes or spectral overlap within the detection window. Our results suggest that for anything but the simplest models, global analysis with physically meaningful models should be used whenever accurate estimates of kinetic parameters are desired to gain important mechanistic insights into the system of interest.

## Materials and Methods

### Ultrafast Spectroscopic Measurements

A detailed description of the femtosecond laser system enabling the ultrafast spectroscopic measurements can be found in our prior publications.^24,36,64^ In brief, the fundamental laser output pulse (FDP) is centered at 800 nm with an average power of ∼3.7 W and a 1 kHz repetition rate. Femtosecond transient absorption (fs-TA) pump–probe measurements of the aforementioned chromophore samples were collected with actinic pump wavelengths of 267 and 400 nm. The 267 nm actinic pump was produced via third harmonic generation (THG) of the FDP using a FemtoKit (EKSMA Optics, UAB). This pulse was not further temporally compressed after THG, resulting in a pulse duration of 200–300 fs. The 400 nm actinic pump was generated through second harmonic generation (SHG) of the FDP, followed by temporal compression using a prism pair (06SB10, Newport, Inc.) to a pulse duration below 100 fs. The average measured pump power ranged from 0.3–0.6 mW. An optical chopper synchronized at half the laser repetition rate was placed into the actinic pump beampath, which enables data collection with the pump on/off repeated sufficient times for a satisfactory SNR (see below). The broadband probe for fs-TA measurements arises from supercontinuum white light generation and the subsequent temporal compression using a chirped mirror pair (DCM-12, 400–700 nm, Laser Quantum, Inc.). The pump and probe pulses were overlapped spatially and temporally onto a 1 mm-thick quartz cuvette (Spectrosil 1-Q-1, Starna Cells, Inc.) that houses the sample of interest, typically in a solution. The beam diameters at the focal point on the cuvette were 150–200 μm. The transmitted probe beam was directed into an imaging spectrograph (IsoPlane SCT-320, Princeton Instruments, Inc.) before being dispersed onto a cooled CCD array camera (PIXIS:100F, Princeton Instruments, Inc.).

The samples were constantly stirred or flowed during the ultrafast spectroscopic measurements to ensure sample stability and limit potential photodegradation during the ∼2 h data collection. For each fs-TA measurement, five-to-six replicate sets including ∼120 preset time points ranging from −2 to 900 ps were collected. At each time point, 3000 laser shots were collected and averaged, representing 1500 pump on/off spectra. This means that during an fs-TA measurement where six replicate sets were collected, each time point represents the average of 9000 total spectra. The precise details regarding sample preparation and optical conditions for fs-TA measurements of catechol,^44^ MPTS,^45^ and phiYFP^46^ in aqueous solution can be found in their respective publications. In general, the organic chromophore sample absorption peak at the laser excitation wavelength has an optical density (OD) of ∼0.5–2.0 per mm.

### Generating Data Sets

Data sets were generated by first creating spectra for each species, simulating the population dynamics for the chosen kinetic parameters, and multiplying them as in eq 1. The spectra were Gaussians with arbitrary amplitudes, center wavelengths, and peak widths. The kinetics were generated using eq S2. If noise was added, Gaussian random numbers were used and the SNR was calculated by dividing the sum of squares of the signal by the sum of squares of the noise.

### Fitting Procedure

Finding a best-fit value for θ was done by minimizing eq 4 using the Levenberg–Marquardt algorithm, implemented the lmfit library.^65^ To make fitting easier, especially since the lifetime parameters can vary by many orders of magnitude, we log-transformed the parameters during the data fitting process. This has the advantage of naturally bounding the variables to be positive, which is true for most of the parameters in the model.^21^ For parameters that could be negative, we shifted it by a lower bound before log transforming. For example, the log-transformed variable corresponding to *t*_0_ was calculated by adding 1 ps, then log-transforming. This step causes the parameter to approach −1 ps as the log-transformed variable approaches minus infinity. Note that we do not log-transform the values in *A*_*i*_(λ), and these are calculated using the “linalg.lstsq” from the SciPy Python library.^66^

To avoid getting trapped in various local minima, fitting was generally done with tens or hundreds of initial conditions chosen at random. Doing this procedure was able to robustly find the best-fit parameters for most models, although the time and number of initial conditions required for a good fit increased as the number of parameters increased. More details on the behavior of the fitting algorithm with the local minima shown are included in the SI Section S1.2 and Figure S1.

### Uncertainty Estimation

Uncertainty estimation was performed by running an MCMC sampling algorithm, starting from the best-fit parameters calculated using the algorithm described above. This was implemented using the “emcee” library in Python, using the stretch-move method.^34^ Note that unlike in a prior report,^7^ here the affine invariance is not necessary since all parameters were log-scaled. We found it to work well empirically. To ensure that the samples were properly sampling the minimum, the change in χ^2^ was monitored during the sampling. If the error decreased during the sampling, this suggested that the MCMC samples were not at the minimum of the χ^2^ surface (see Figure S2a for example). The procedure was repeated until the χ^2^ values during the sample did not noticeably decrease (see Figure S2b for example). The probability distribution was calculated only after subsampling by the autocorrelation time computed with the “get_autocorr_time” function. See the SI Sections S1.3 and S1.4 as well as Figures S2 and S3 for more details.

## Supplementary Material



## Data Availability

The data sets with analysis and discussions supporting this article have been incorporated and presented in the main text and SI. The computational code and spectral data sets (on catechol, MPTS, and phiYFP) used to generate all figures for this paper are publicly available through GitHub (located at https://github.com/sully-bd/spectroscopy_error_analysis). The code is currently not written to be an installable package, since it does not include some functionalities that would allow it to readily handle general experimental data sets. Mainly, it cannot handle complex or significant optical artifacts around time zero. We welcome future development by others or incorporating the ideas presented herein (including the main text and SI) into widely used packages.

## Notes

Text on experimental, nonlinear fitting, and MCMC sampling methods, nonlinear fitting and error analysis examples with various initial parameters, local minima, approaching lifetimes, different number of wavelengths, and a reduced signal-to-noise ratio (Figures S1–S5), as well as additional references (PDF)

## †

Department of Physics, Cornell University, 109 Clark Hall, Ithaca, New York 14853, United States
