# Supplementary material for: Characterization of Parameter Uncertainty in Global Analysis for Ultrafast Spectroscopy Using Markov Chain Monte Carlo Sampling
Source: Precis Chem. 2026 Mar 27;4(5):674–89. doi: 10.1021/prechem.5c00468 (PMC13217343; doi:10.1021/prechem.5c00468)
Supplement: Supplementary file 1 [file pc5c00468_si_001.pdf]

Supporting Information (SI) for

Characterization of Parameter Uncertainty in Global Analysis for Ultrafast Spectroscopy using Markov Chain Monte Carlo Sampling

Sullivan Bailey-Darland,[†] Logan S. Lancaster, Taylor D. Krueger, Cheng Chen and Chong Fang*

Department of Chemistry, Oregon State University, 153 Gilbert Hall, Corvallis, Oregon 97331 USA

Corresponding author e-mail: Chong.Fang@oregonstate.edu (C. Fang).

Web: https://fanglab.oregonstate.edu/

[†]Current address: Department of Physics, Cornell University, 109 Clark Hall, Ithaca, NY 14853

Table of Contents

S1. Supplementary Text S3

S1.1 Calculation of population dynamics S3

S1.2 Nonlinear fitting details S3

S1.3 Derivation of probability from the cost function S5

S1.4 MCMC sampling S7

S1.4.1 Two approaching lifetimes with sequential kinetics S9

S1.4.2 Varying the number of wavelengths used in fitting the catechol data S9

S1.4.3 Details for the yellow fluorescent protein chloride sensor kinetics S9

S2. Supplementary Figures S11

Figure S1. Representative examples for local minima after the nonlinear fitting procedure. S11

Figure S2. Effects of the initial parameter selection on the minimization route. S12

Figure S3. Error analysis for two approaching lifetimes that follow the sequential kinetics. S13

Figure S4. Effects of varying the number of wavelengths used for fitting on the uncertainties of retrieved parameters from the acidic catechol in water following a three-component sequential model. S14

Figure S5. Error analysis of the transient absorption spectra of catechol in acidic (pH 4) buffer after 267 nm excitation with a much reduced signal-to-noise ratio. S15

S3. Supplementary References S16

S1. Supplementary Text

S1.1 Calculation of population dynamics

For completeness, we describe additional details on the fitting procedure used in this work. As mentioned in main text, the population dynamics for first-order kinetics with a Gaussian instrument response function (IRF, given by Eq. 3) has been solved analytically;[1] we state the result herein without explicit derivation. For a choice of kinetic parameters, the transfer matrix T_{ij} and IRF are fully defined. The population dynamics are computed by first calculating the eigendecomposition of the transfer matrix $T = U\Lambda U^{-1}$, where U is the matrix of eigenvectors and Λ is a diagonal matrix of eigenvalues. The population dynamics are then given by

$$X_i(t) = \sum_{j,k} U_{ij} \left(e^{-\Lambda_{jj}t} * \mathrm{IRF}(t)\right) U_{jk}^{-1} J_k \tag{Eq. S1}$$

where $*$ represents a convolution. The convolution between a Gaussian function (the IRF) and an exponential decay can be calculated analytically as

$$e^{-kt} * \mathrm{IRF}(t) = \frac{1}{2} e^{-kt} \exp\left(k\left(t_0 + \frac{\sigma^2 k}{2}\right)\right)\left[1 + \mathrm{erf}\left(\frac{t - k(t_0 + \sigma^2)}{\sigma\sqrt{2}}\right)\right] \tag{Eq. S2}$$

These equations were implemented in NumPy (Numerical Python)[2] and vectorized for speed.

S1.2 Nonlinear fitting details

Here we present more details on the nonlinear fitting method used to find the best-fit kinetic parameters. As described in main text we used the Levenburg-Marquardt algorithm from a large

number of initial parameter choices. We found this was very important to avoid getting stuck in a non-optimal local minimum, and the number of initial choices required scaled with the difficulty of the fitting problem (*e.g.*, number of species, amount of noise). This was true for both the generated and experimental datasets.

For a given dataset and kinetic model, we drew kinetic parameters independently and uniformly between a set of bounds. As was described in main text, we used log-transformed parameters since the parameters often varied by many orders of magnitude. For example, if the parameters were not log-transformed the uniform sampling would yield lifetimes that are all on the order of microseconds. The natural-log-transformed parameters were chosen at random, uniformly between 0 and 8. For lifetimes, this corresponds to the range between 1 fs and ~3 ps. We found this worked well empirically, but this could likely be optimized further.

For each of the randomly chosen sets of parameters (initial starts), the χ^2 function was minimized *via* the Levenburg-Marquardt algorithm implemented using "lmfit" with a maximum of 1,000 iterations to reach an optimized set of parameters. Generally, tens or hundreds of initial choices were used. The parameters with the lowest χ^2 value were used as the best-fit result. This procedure usually took a few minutes on a laptop computer, although it was slower as the models became larger and the size of the datasets increased (see relevant examples and discussions in main text). The structure of the optimized χ^2 values tended to show a variety of local minima reached by the minimizer. Some examples are shown below in Figure S1, where the final χ^2 values after the Levenburg-Marquardt minimization are shown for 150 attempts. In general, the majority of optimization runs do not reach the best-fit parameters and instead reach a variety of other fits. We interpret the step-like pattern as demonstrating the existence of a few local minima that some fraction of the fitting attempts get trapped in. Empirically, this finding could explain the difficulty

in fitting procedures that some users or researchers experience even for spectral datasets with a decent signal-to-noise ratio (SNR, see Figure S1b,c for example).

S1.3 Derivation of probability from the cost function

As described in main text (see Eq. 5), the probability function we used was a function of only the kinetic parameters. These are not the only parameters in the model, though, since the species spectra are all adjusted to fit the data. For this work we were not interested in the uncertainty in those parameters and would like to only study the uncertainty in the kinetic parameters θ.

There is a subtlety involved in ignoring the A parameters. Note that A refers to all the parameters $A_n(\lambda_j)$, covering each species and measured wavelength. Since they are also parameters in the model, it may be more accurate to write $P(\theta, A|D) \propto P(D|\theta, A)\,\pi(\theta, A)$ in place of Eq. 5. These parameters are then considered nuisance parameters. Since we are not necessarily interested in their distribution, the most common practice is to integrate them out.[3] Intuitively, the likelihood of the kinetic parameters depends on the likelihood of the corresponding spectral parameters. If the best-fit spectra for a choice of kinetic parameters are all very improbable, then the kinetic parameters are also improbable. Performing this integration and splitting the prior gives the following expression:

$$P(\theta|D) \propto \pi(\theta) \int dA\, P(D|\theta, A)\, \pi(A|\theta) = \pi(\theta) \int dA\, N\, \mathrm{e}^{-\frac{1}{2}\chi^2(\theta,A)}\, \pi(A|\theta)$$

(Eq. S3)

There are then a few options to evaluate this quantity, depending on the choice of the prior $\pi(A|\theta)$. First, we can consider that A assumes the optimal value for each θ. This can be written mathematically as $\pi(A|\theta) = \delta(A - A^*)$ with $A^* = A^*(\theta)$ being the best-fit choice of A for a given θ. Using this in Eq. S3 yields Eq. 5. Another option is to perform the integral with a uniform

prior. Adapting the procedure in Brown *et al.*,[4] the function $\chi^2(\theta, A)$ can be expanded around its minimum for all the spectral parameters. This expansion is exact, since the parameters appear in the fit linearly (and in the error quadratically). Performing the expansion yields

$$\chi^2(\theta, A) = \chi^2(\theta, A^*(\theta)) + \frac{1}{2}\sum_n \sum_j \left(\frac{\partial^2 \chi^2}{\partial A_n(\lambda_j)^2}\right)_{\{\theta, A^*(\theta)\}} \left(A_n(\lambda_j) - A_n^*(\lambda_j)\right)^2$$

$$= \chi^2(\theta, A^*(\theta)) + \sum_i \left(\frac{X_n(t_i)}{\sigma_{ij}}\right)^2 \left(A_n(\lambda_j) - A_n^*(\lambda_j)\right)^2$$

(Eq. S4)

Using this expression in the integral, with a uniform prior, gives the probability distribution as

$$P(\theta|D) \propto \pi(\theta) e^{-\frac{1}{2}\chi^2(\theta, A^*(\theta))} \prod_{n,j} \sqrt{\frac{2\pi}{\sum_i \left(\frac{X_n(t_i)}{\sigma_{ij}}\right)^2}} = P(\theta, A^*(\theta)|D) \prod_{n,j} \sqrt{\frac{2\pi}{a_{nj}(\theta)}}$$

(Eq. S5)

where $a_{nj}(\theta)$ is roughly describing the impact of changes in $A_n(\lambda_j)$ to the fit. If $a_{nj}(\theta)$ is very small then $A_n(\lambda_j)$ has little effect on the fit. However, this formula quickly runs into a problem. The probability can be maximized by having $a_{nj}(\theta) \to 0$, which can be accomplished by having some of the species populations go to zero. Numerically, we found that running a sampling or fitting routine resulted in a fit which chose unrealistic kinetic parameters so that $a_{nj}(\theta) \to 0$ and the probability diverged.

The issue with the uniform prior shows that there must be some constraints on the allowed values for A. The simplest method, and what was also used in a prior study[5] when they required an integrable prior, is to use a Gaussian prior to constrain A near A^*, *i.e.*, $\pi(A|\theta) = \prod_{n,j} \frac{k}{\sqrt{2\pi}} e^{-\frac{1}{2}k\left(A_n(\lambda_j) - A_n^*(\lambda_j)\right)^2}$ where the choice of k determines the width of the Gaussian profile.

However, in practice this seemed to have relatively little effect compared to fixing A to A^*. For simplicity, in this work we fixed A to A^* (*i.e.*, the best-fit choice of A for a given θ). Future work may want to consider other choices of the prior, to include physical constraints; for example, one could choose a prior to require the spectra to be positive, negative, smooth, etc.

S1.4 MCMC sampling

The goal for MCMC sampling is to estimate the probability distribution $P(\theta|D) = N\,\mathrm{e}^{-\frac{1}{2}\chi^2(\theta)}\pi(\theta)$, since this equation is difficult to use in practice without knowing the normalization factor *N*. One might consider calculating N through integrating: $1/N = \int d\theta\,\mathrm{e}^{-\frac{1}{2}\chi^2(\theta)}\pi(\theta)$. Computationally this is not a good method for a few reasons. The number of points scales exponentially in the number of parameters, and the vast majority of the points contribute almost nothing to the integral (due to the exponential decay structure). This results in the value of the integral depending only on a small region in parameter space.

A common method for estimating probability distributions that are difficult to calculate analytically is Markov chain Monte Carlo (MCMC) sampling. The first application was in calculating the normalization for a partition function,[6] but has been used widely since. This method was later suggested by Holzwarth,[7] but was discarded due to high computational costs at the time. The basic idea is to generate samples by taking random steps, and accepting or rejecting the steps based on the probability distribution. The original Metropolis-Hastings algorithm rule was to accept a step from θ_1 to θ_2 with probability

$$P_{\mathrm{step}}(\theta_1 \to \theta_2) = \min(P(\theta_2)/P(\theta_1), 1) \qquad \text{(Eq. S6)}$$

In other words, the step is guaranteed if the new parameters are more likely, but it is still possible if the likelihoods of the two states are not so different. It can be shown[6] that samples generated using this rule would act as if they were generated from the probability distribution $P(\theta)$. It is notable that because only the ratio of $P(\theta)$ appears, the normalization is not needed to calculate this probability.

As mentioned in main text, there are a few technical details when performing MCMC sampling, mainly due to the fact that the samples are only guaranteed to approach the true distribution as the number of samples goes to infinity (and we cannot do this in practice). Since the method is designed to randomly explore, it is not well-designed to find a minimum in $\chi^2(\theta)$; it is thus best to start the sampling from a best-fit value found through a more traditional minimization routine. If this is not the case, the MCMC sampling will have to first find the area near the minima, which can be seen by a decrease in the χ^2 values during the sampling.

A brief demonstration is shown, using the dataset from Figure 1c,d (the case for SNR of 20). We display the χ^2 values from MCMC sampling routine starting from a randomly chosen parameter set without any nonlinear minimization (Figure S2a). A properly equilibrated sampling is shown in Figure S2b, where the optimization procedure described in Section S1.2 was conducted first. This issue can also be alleviated by allowing the system to "thermalize" first, by running some number of steps before using them to estimate $P(\theta)$. For example, we could start the MCMC sampling routine again from the best-fit sample, originally from the non-thermalized case.

The other technical details involve the method of choosing random steps and ensuring that samples are decorrelated from each other. In short, sampling with completely random steps tends to explore the space relatively slowly, and causes the steps to be highly correlated with each other. Efficiently choosing steps and using only steps that are sufficiently decorrelated can significantly

speed up the convergence to $P(\theta)$. Both of these challenges are addressed in the Python "emcee" library, which has been used in this work to generate samples to estimate $P(\theta)$.[8]

S1.4.1 Two approaching lifetimes with sequential kinetics

In main text, the χ^2 surface and MCMC samples for two exponential decays in parallel were shown as the two lifetimes approach each other (see Figure 2a to d). Here, we demonstrate the same results for a sequential model (Figure S3). Unlike the parallel case, the parameter distributions do not extend to infinity or zero as the lifetimes become similar. This is because a single exponential cannot accurately fit the kinetics for a sequential model.

S1.4.2 Varying the number of wavelengths used in fitting the catechol data

As described in main text, the parameter certainty for kinetic parameters shown in Figure 5 depends strongly on the number of wavelengths measured: decreasing the number of wavelengths used would reduce the parameter certainty. For corroboration, we present the kinetic parameter distributions as a function of the number of wavelengths used for fitting (Figure S4). The fitting procedure and underlying kinetic model are otherwise identical to what was used for Figure 5. Moreover, the effects of a much reduced SNR were examined in Figure S5 below, showing the comparative data plots of global analysis with error analysis *vs.* the high SNR case in Figure 5.

S1.4.3 Details for the yellow fluorescent protein chloride sensor kinetics

The kinetic model used for fitting the phiYFP data (shown in Figure 7 in main text) was taken from the model proposed in our prior report,[9] with some additional components added to fit the coherent artifacts near the time zero of photoexcitation, as well as having less restraints for the

bifurcated pathways after the molecular system moves out of the initial Franck-Condon region. The kinetic rate matrix used is listed below:

$$\begin{pmatrix} k_1 & 0 & 0 & 0 & 0 & 0 & 0 & 0 & 0 \\ 0 & k_2 & 0 & 0 & 0 & 0 & 0 & 0 & 0 \\ 0 & 0 & 0 & k_3 & 0 & 0 & 0 & 0 & 0 \\ 0 & 0 & 0 & 0 & k_4 & 0 & k_5 & 0 & 0 \\ 0 & 0 & 0 & 0 & 0 & k_6 & 0 & 0 & 0 \\ 0 & 0 & 0 & 0 & 0 & k_7 & 0 & 0 & 0 \\ 0 & 0 & 0 & 0 & 0 & 0 & 0 & k_8 & 0 \\ 0 & 0 & 0 & 0 & 0 & 0 & 0 & 0 & k_9 \\ 0 & 0 & 0 & 0 & 0 & 0 & 0 & 0 & k_{10} \end{pmatrix}$$

and the vector J_i describing the initially excited population was $(1 \quad 1 \quad 1 \quad 0 \quad \cdots \quad 0)$. In this matrix, the transition from **i** to **j** is given by the off-diagonal element in row **i** and column **j**, while the diagonal elements indicate a direct decay to the ground state. The first two species (k_1 and k_2 with self-decay) were used to fit the IRF, and the third species is the initially excited population that transitions to the rest of the model (k_3 that denotes species 3 going to species 4, which is shown as state 1 post IRF in Figure 7a).[9] These early spectra are not shown in the main text (Figure 7b) since they mainly capture unphysical features from the IRF. All the subsequent rate constants (*i.e.*, k_4 to k_{10}) can be converted to the corresponding time constants with their error analysis shown in Figure 7c. Again, we found that the error bar increases if the wavelengths are sub-sampled, reminiscent of Figure S4 below.

S2. Supplementary Figures

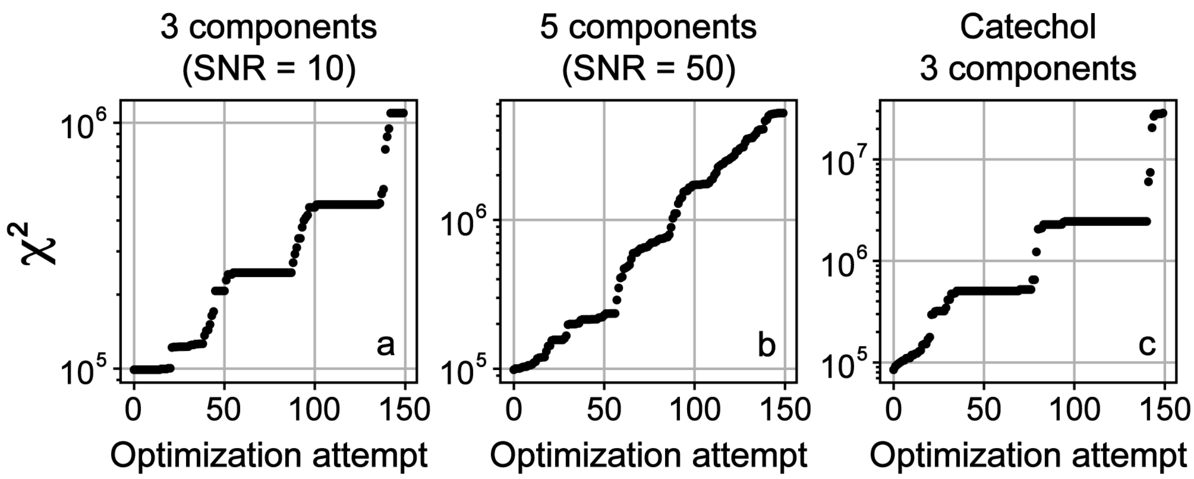

Figure S1. Representative examples for local minima after the nonlinear fitting procedure. The χ^2 values after Levenburg-Marquardt minimization for (a) a three-component generated dataset with a signal-to-noise ratio (SNR) of 10, (b) a five-component generated dataset with an SNR of 50, and (c) the catechol dataset analyzed in Figure 5 as shown in main text. The optimization attempts were sorted from the lowest to highest χ^2 values before plotting in each panel.

We note that each attempt was a separate optimization run, standard from a different initial condition. This procedure was performed to find the global minimum, since many choices of initial parameters could get trapped in various local minima, as shown in Figure S1 above (also detailed in Section S1.2). The plateaus correspond to distinct local minima (that a large number of initial conditions get trapped in), which become less significant with a higher SNR as demonstrated for this dataset and model (see Figure S1b). The χ^2 value on the left side (*i.e.*, the smallest one) corresponds to the best guess at the global minimum in each case/panel.

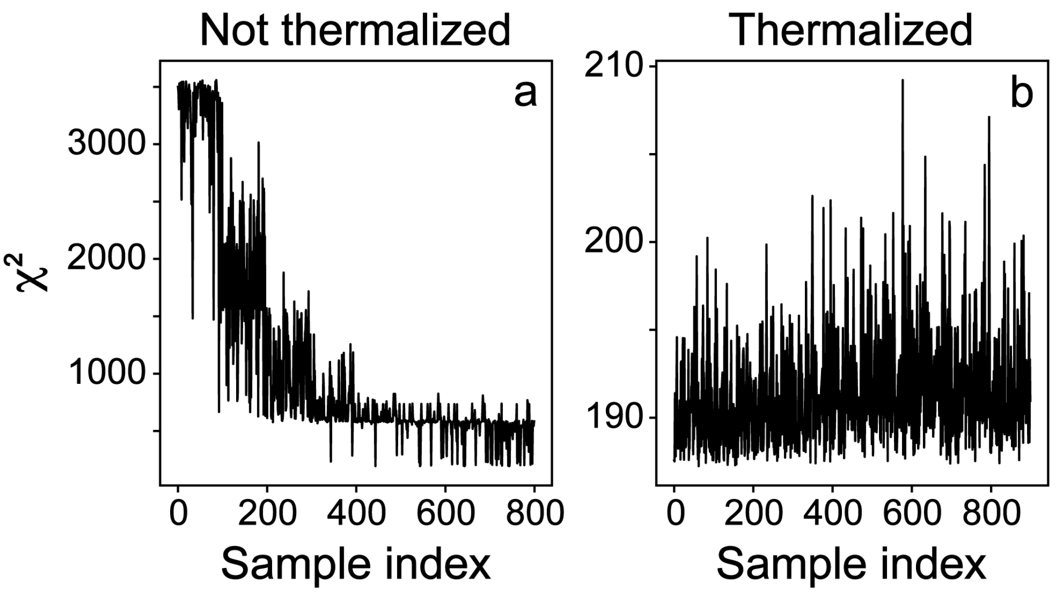

Figure S2. Effects of the initial parameter selection on the minimization route. The χ^2 values from MCMC sampling for the generated dataset from Figure 1c,d (SNR of 20), for (a) a random choice of parameters and (b) best-fit parameters found using the optimization procedure described in Section S1.2. The χ^2 values for the random choice decrease during the sampling, indicating the samples are not at "thermal" equilibrium and are still relaxing to the minimum (see left panel). The χ^2 values for the optimized parameters do not trend in any direction, so they are "thermalized" and an accurate estimate of the probability distribution (see right panel).

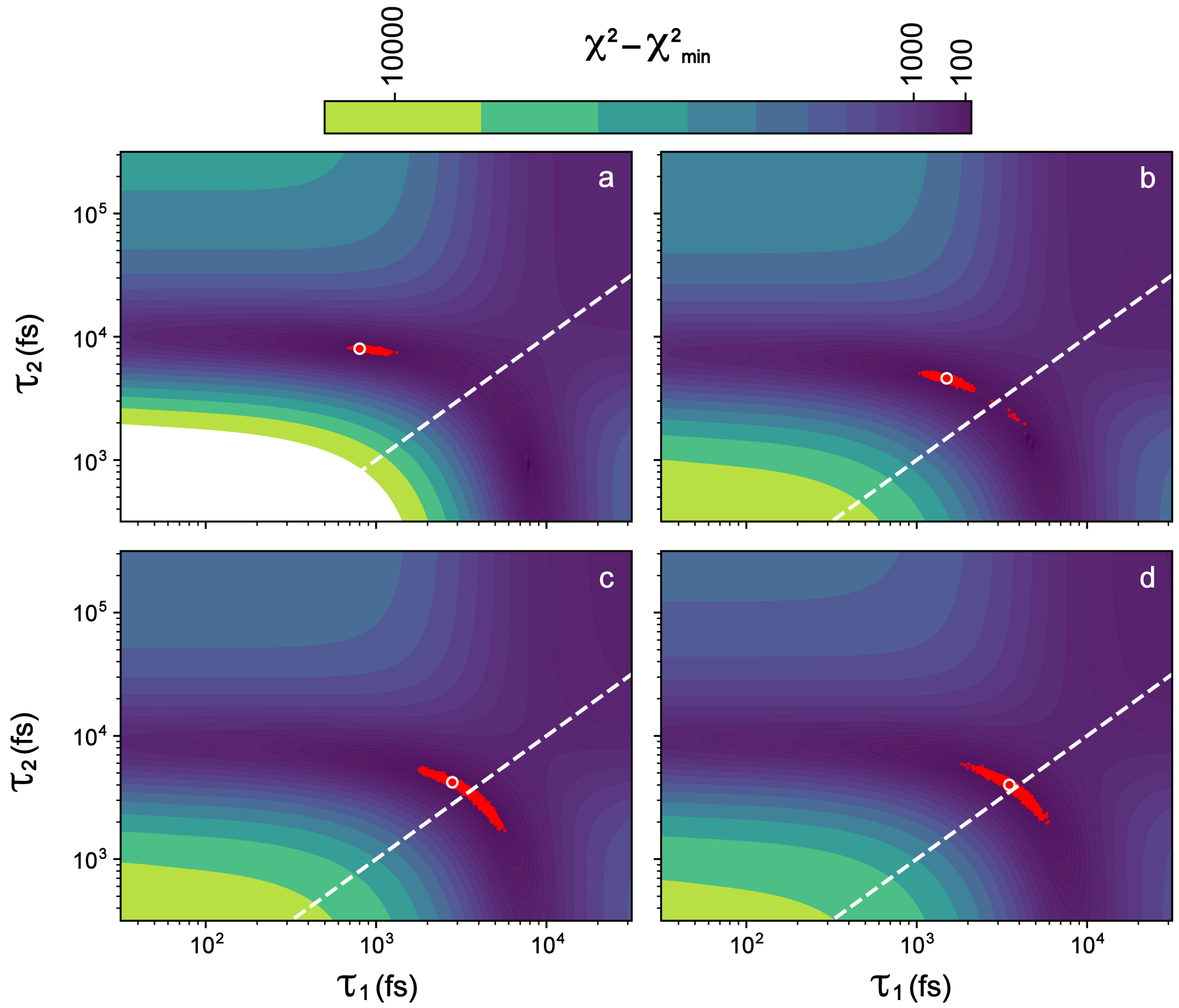

Figure S3. Error analysis for two approaching lifetimes that follow the sequential kinetics. The χ^2 surfaces as a function of the lifetimes (with IRF parameters fixed) when fitting biexponential decays with added noise as the lifetimes become increasingly similar, for sequential kinetics. The lifetimes and noise used are identical to Figure 2 as shown in main text, although the lifetimes are not constrained to the $\tau_1 \leq \tau_2$ condition in this figure. The white hollow circles denote true parameters (panel a, 0.8 and 8 ps; panel b, 1.5 and 4.6 ps; panel c, 2.8 and 4.2 ps; and panel d, 3.5 and 4 ps) while the dashed lines denote $\tau_1 = \tau_2$ (which are the farthest and closest to selected parameters in panels a and d, respectively). The MCMC samples generated for this dataset, which approximate the probability distribution of the aforementioned parameters, are depicted as red dots.

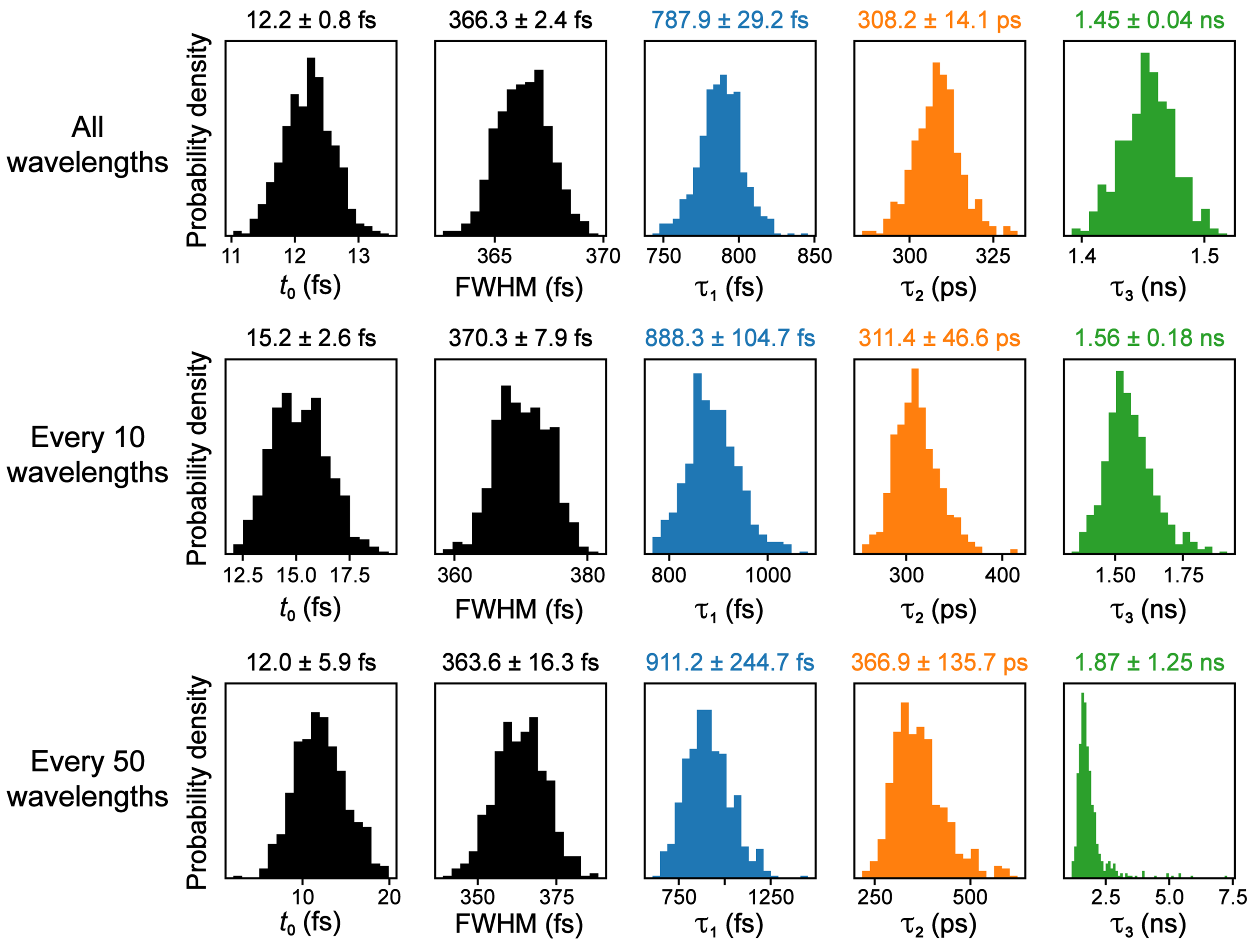

Figure S4. Effects of varying the number of wavelengths used for fitting on the uncertainties of retrieved parameters from the acidic catechol in water following a three-component sequential model. The top row shows the probability distributions for all wavelengths (1340 wavelength points), the middle row is fitting with every 10 wavelengths (134 points), and the bottom row is fitting with every 50 wavelengths (27 points). As the number of wavelengths used decreases, the uncertainty in the retrieved parameters increases (as reflected by the error bars listed above each panel plot, both in absolute values and percentages, becoming larger from top to bottom panels). The parameter distributions for the longest lifetimes (rightmost panels) become more skewed with less data points for the fitting analysis.

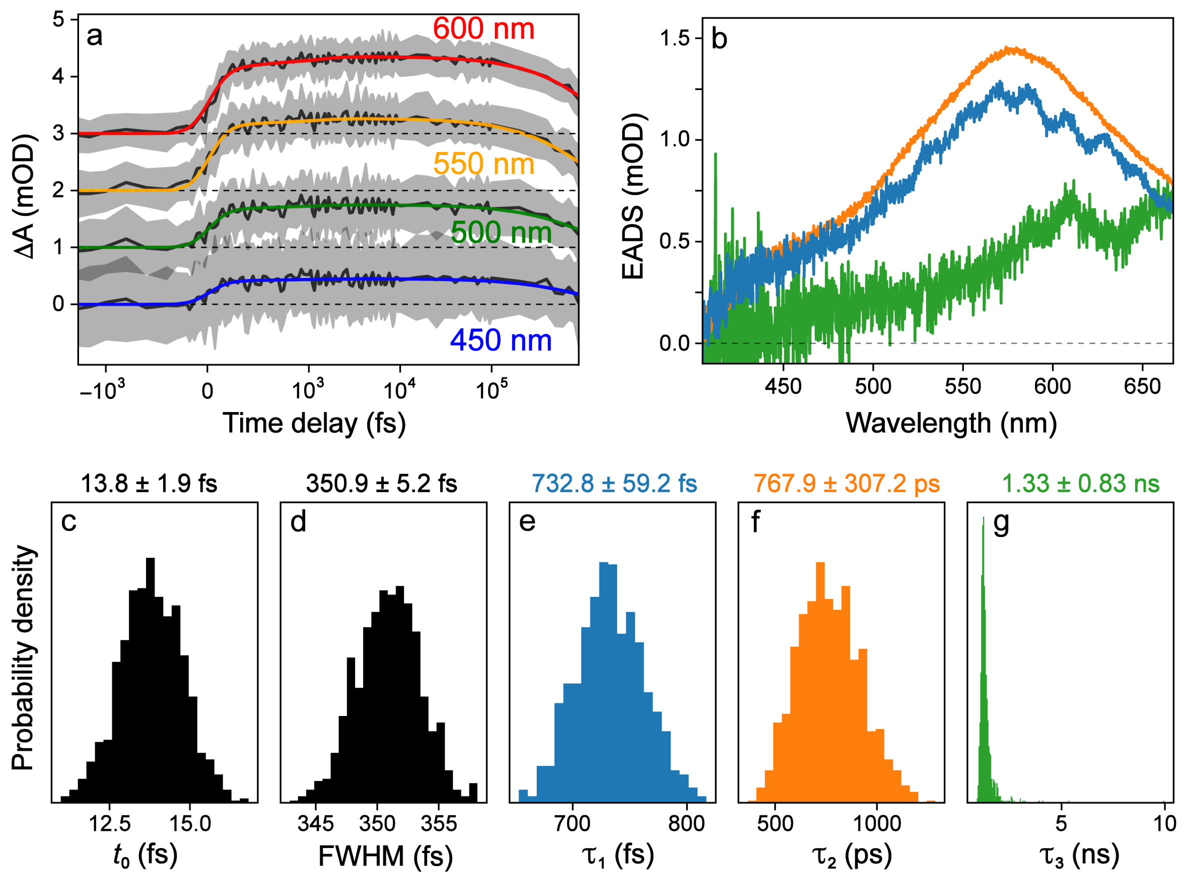

Figure S5. Error analysis of the transient absorption spectra of catechol in acidic (pH 4) buffer after 267 nm excitation with a much reduced signal-to-noise ratio. Fits from global analysis are shown (a) at several probe wavelengths denoted in the insets (the color-coded solid traces denote the global fits achieved across the spectral range while the gray shade shows the noise level, which is much larger than the counterpart in Figure 5a), along with (b) the resulting evolution-associated difference spectra (EADS, color-coded) for each species/states. (c-g) The probability density distributions of kinetic parameters are shown as histograms, calculated from MCMC sampling. Notably, since we only used one experimental dataset instead of the average of all five datasets for this "control" analysis, the noise should increase by a factor of ~2.2 (*i.e.*, $\sqrt{5}$) which leads to the larger error bars (as expected) being denoted above all the histograms in panels c–g *vs.* Figure 5c–g. In essence, the MCMC framework and uncertainty estimates remain reliable and informative.

S3. Supplementary References

(1) Slavov, C.; Hartmann, H.; Wachtveitl, J. Implementation and Evaluation of Data Analysis Strategies for Time-Resolved Optical Spectroscopy. *Anal. Chem.* **2015**, *87* (4), 2328–2336.

(2) Harris, C. R.; Millman, K. J.; van der Walt, S. J.; Gommers, R.; Virtanen, P.; Cournapeau, D.; Wieser, E.; Taylor, J.; Berg, S.; Smith, N. J.; *et al.* Array programming with NumPy. *Nature* **2020**, *585* (7825), 357–362.

(3) Berger, J. O.; Liseo, B.; Wolpert, R. L. Integrated likelihood methods for eliminating nuisance parameters. *Statist. Sci.* **1999**, *14* (1), 1–28.

(4) Brown, K. S. Signal transduction, sloppy models, and statistical mechanics (Ph.D. thesis). Cornell University, 2003. https://catalog.library.cornell.edu/catalog/8034254.

(5) Brown, K. S.; Sethna, J. P. Statistical mechanical approaches to models with many poorly known parameters. *Phys. Rev. E* **2003**, *68* (2), 021904.

(6) Metropolis, N.; Rosenbluth, A. W.; Rosenbluth, M. N.; Teller, A. H.; Teller, E. Equation of State Calculations by Fast Computing Machines. *J. Chem. Phys.* **1953**, *21* (6), 1087–1092.

(7) Holzwarth, A. R. Data Analysis of Time-Resolved Measurements. In *Biophysical Techniques in Photosynthesis*, Amesz, J., Hoff, A. J. Eds.; Advances in Photosynthesis and Respiration, Springer Dordrecht (Netherlands), 1996; pp 75–92.

(8) Foreman-Mackey, D.; Hogg, D. W.; Lang, D.; Goodman, J. emcee: The MCMC Hammer. *Publ. Astron. Soc. Pac.* **2013**, *125* (925), 306–312.

(9) Chen, C.; Tutol, J. N.; Tang, L.; Zhu, L.; Ong, W. S. Y.; Dodani, S. C.; Fang, C. Excitation ratiometric chloride sensing in a standalone yellow fluorescent protein is powered by the interplay between proton transfer and conformational reorganization. *Chem. Sci.* **2021**, *12* (34), 11382–11393.
